# Nourished, Exposed Beaches Exhibit Altered Sediment Structure and Meiofaunal Communities

**DOI:** 10.3390/d12060245

**Published:** 2020-06-15

**Authors:** Stephen R. Fegley, Julian P. S. Smith, Douglas Johnson, Amelia Schirmer, Jeremiah Jones-Boggs, Austin Edmonds, Joseph Bursey

**Affiliations:** 1 Institute of Marine Sciences, University of North Carolina at Chapel Hill, Morehead City, NC 28557, USA;; 2 Department of Biology, Winthrop University, Rock Hill, SC 29733, USA;; 3 Department of Biology, Barton College, Wilson, NC 27893, USA;

**Keywords:** meiofauna, beach nourishment, beach sedimentary structure

## Abstract

To retain recreational uses and shoreline protection, a large proportion of ocean beaches have been, and continue to be, nourished. Adding sand from subtidal and terrestrial sources to nourish beaches rarely re-creates the original sediment structure of the beach. Numerous studies have demonstrated that meiofaunal communities are altered by changes in sediment composition in low-energy substrates, therefore, we have explored whether beach nourishment has affected exposed, ocean beach meiofaunal communities. Since the early 2000s, we have conducted a series of sampling and experimental studies on meiofauna and sediments on nourished beaches in coastal North Carolina USA that had been sampled previously in the early 1970s, prior to any beach nourishment. Most of our studies consider meiofauna at the level of major taxa only. However, a few studies examine free-living flatworm (turbellarian) species in detail because of the existence of historical studies examining this group. Comparison of contemporary results to historical data and of heavily nourished versus lightly nourished beaches reveals extensive changes to beach sediment structure and meiofaunal community composition, indicating that the beaches are a more heterogeneous habitat than in the past. The effects of these substantial physical and biological changes to the production of beach ecosystem services are unlikely to be inconsequential.

## 1. Introduction

Beach nourishment has served as a popular mitigation practice globally, and especially along the United States Atlantic and Gulf of Mexico coasts, to counter beach erosion [1]. This practice, which involves placing sediment (the fill) dredged or mined from other locations onto or near eroded beaches, has been viewed as having several benefits [2]. Among the presumed benefits, it avoids unwanted but unavoidable consequences of hardening exposed shorelines [3]. Nourishment also provides an opportunity to dispose of spoil removed from shipping channels and marinas [4]. In addition, some argue that it creates a habitat for beach fauna and flora that depend on the presence of sediments along sandy shorelines [5,6]. Although the use of nourishment is being questioned recently for economic and risk-related issues [7–9], the first two benefits often hold true, but the last has been and is highly controversial.

Numerous studies have demonstrated both short-term (days to weeks) and long-term (months to years) negative consequences of beach nourishment to a majority of the flora and fauna that inhabit or utilize the backbeach, forebeach, and contiguous nearshore beach habitats [1,3,10–18]. During and immediately after nourishment, abundances of terrestrial and marine invertebrates, surf-zone fish, and birds plummet due to both direct effects (e.g., smothering or change in sediment composition) or indirect effects (loss of prey, alteration of beach profile, darkening of beach color, or changes in water quality) associated with beach nourishment. Unfortunately, even if beach communities recover, continued beach loss and the need for subsequent beach restoration by nourishment will occur because continued sea level rise and nearshore development promote persistent beach erosion [7]. A lack of beach equilibrium produces a scenario where beach habitats may be perennially altered from their historical condition.

The numerically dominant metazoans living in exposed beach habitats are not readily observed because they are small (micrometers to a few cm’s in size) and infaunal [18]. Meiofauna, metazoans ranging from 50–500 μm in length, occur in exposed beaches in high abundances (>10,000 individuals under each m^2^ of beach) and high diversity (>200 species under each m^2^ of beach). Extracting and identifying these organisms has challenged investigation of the functional role of this contingent of the beach community. Meiofauna serve as prey and, more importantly, influence the microbial communities in the beach that are involved in the essential ecoservice of recycling nutrients in coastal waters [19]. In many habitats, including exposed beaches, the structure of meiofaunal communities or distribution of individual species have been demonstrated to be sensitive to sediment composition [20]. This raises the question of whether changes to sediment composition within exposed beaches, subjected to nourishment, have affected resident meiofaunal community structure and, potentially, any meiofauna-mediated processes associated with beach ecosystem function.

We report on a collection of studies conducted independently over an interval of ~14 years, all of which were constrained by the goal of providing insight into whether exposed beach sediments and the associated meiofaunal assemblages differ depending on time (spanning decades) or location (alongshore) within a barrier island. These studies were prompted by the existence of studies from the late 1960s through to the mid-1970s, that we could repeat in the same locations of the same beaches during the 2000s. This interval is important because the historical studies were conducted prior to the use of beach nourishment on all the sampled beaches. However, we determined that additional contemporary studies were required to contextualize the results of repeating historical studies and to fully characterize sediments and meiofauna on these beaches (Table 1). Our specific questions are: (1) is there evidence that site-specific beach sedimentology and meiofaunal community structure have changed between the pre- and post-nourishment eras?; (2) how does beach sedimentology and meiofaunal community structure differ among beaches currently?; (3) do small-scale (cm’s to m’s) spatial differences in sedimentology horizontally and vertically predict meiofaunal abundances?; and (4) do species-level sedimentology-faunal patterns reveal any differences to major taxon-level patterns?

## 2. Materials and Methods

### 2.1. Study Area

**Figure 1.**
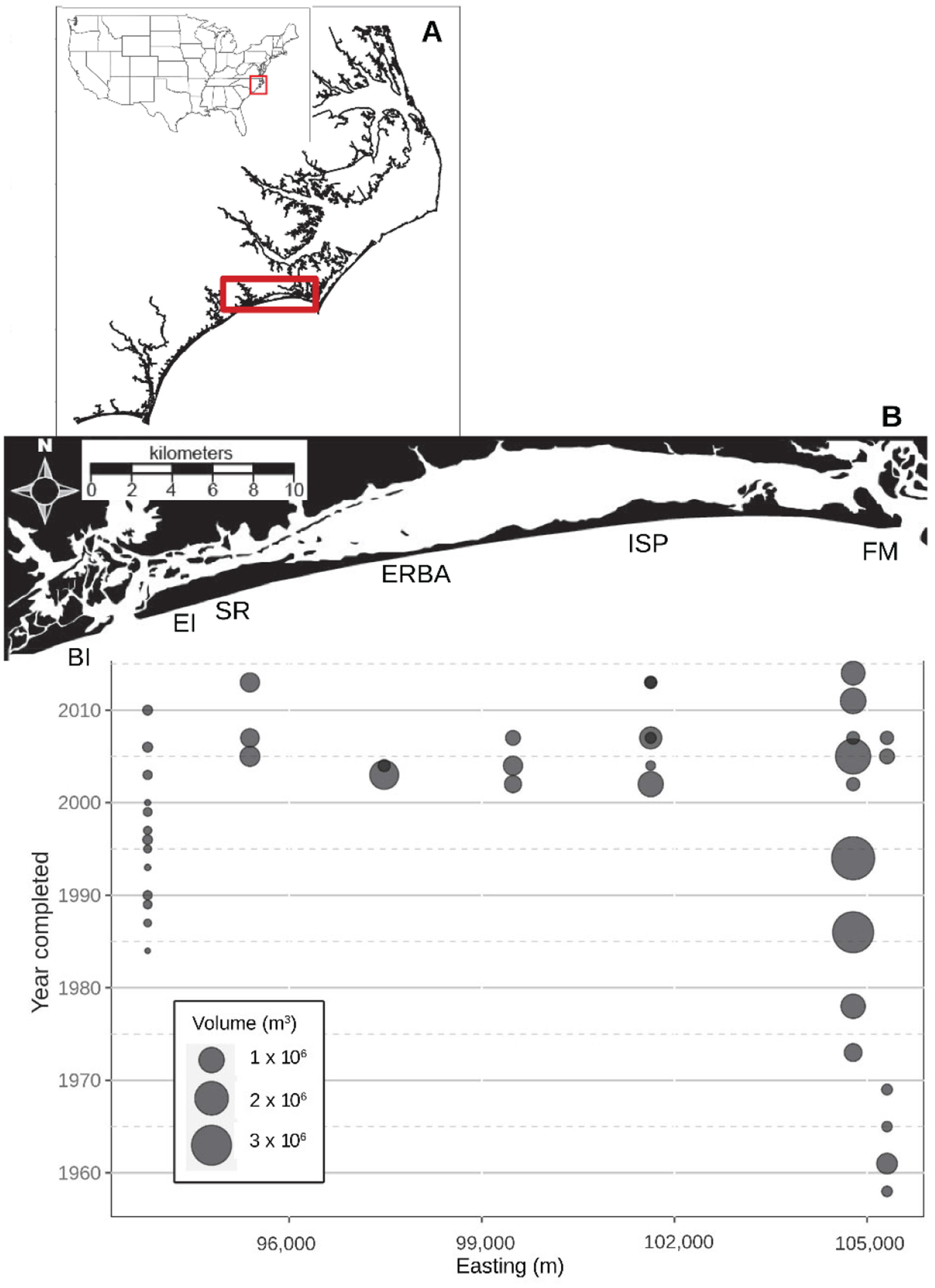
Location of Bogue Banks, North Carolina, USA **(A)** and the nourishment history of the island **(B)**. The bubbles on the bubble plot below indicate the approximate midpoint of the spoil area for each recorded nourishment event as well as the approximate volume (m^3^) of material emplaced. Study beaches referred to in the text: BI—Bear Island; EI—Emerald Isle; SR—Spinnaker’s Reach; ERBA—Eastern Regional Beach Access; ISP—Iron Steamer Pier; and FM—Fort Macon.

A majority of the studies described below were conducted on exposed beaches occurring on a barrier island called Bogue Banks located on the southeastern North Carolina, USA coast (Figure 1). The characteristics of these beaches have been described in greater detail elsewhere [10,21]. Briefly, these microtidal (~1 m) beaches experience waves ranging from 1–2 m in height except during storms. In most locations, the backbeach fronts onto dunes ranging from 1–4 m in height. Beyond the dunes there is extensive commercial and residential development. The dominant longshore drift is from east to west along the south facing beaches. Beach widths range from 25–100+ m. The typical beach profile has a flat (<1°) backbeach extending from the dune to roughly halfway to the subtidal. The forebeach consists of a steeper (~4–7°) high/mid intertidal and less steep (<4°), wider, low intertidal. Sediments are dominated by fine to medium quartzite sands with localized concentrations of carbonate particles and heavy minerals.

Bear Island (BI) located on the barrier island immediately west of Bogue Banks, was sampled in 2017. Several years prior, a localized, volumetrically small single beach nourishment occurred there. This location offered an exposed beach, unaffected directly or indirectly by large volumes of beach nourishment sediments and disturbances, that experiences comparable hydrodynamic and climatic conditions to the neighboring Bogue Banks beaches. Consequently, it served as an ideal reference site with no to little nourishment compared to Bogue Banks.

### 2.2. Nourishment History

Prior to 2000, beach nourishment was limited to the easternmost and westernmost ends of Bogue Banks (Figure 1). These were relatively small (<1 × 10^6^ m^3^) disposals of sediment dredged from nearby inlets or, in the case of Fort Macon (FM) State Park beach, the Morehead City port basin. In response to continuing erosion of the FM beach, much larger volumes of sand were deposited on the eastern end of the island starting in the mid-1970s and continuing to this day. On the westernmost point of Bogue Banks, at a location ~1.5 km west of Spinnaker’s Reach (SR), beach disposals of Bogue Inlet sediments were placed frequently on the beach to protect homes abutting the inlet beach.

During the 2000s, a multi-year project emplaced multiple large (>1 × 10^6^ m^3^) volumes of sediments on beaches along the entire length of Bogue Banks (Figure 1). Some beaches were nourished repeatedly during the decade. Numerous studies documented the effects of this specific nourishment project on sediment characteristics, beach macrofauna, shorebirds, and surf fish [10,12,14,15,22,23]. Since the end of that project in 2007, sediment disposal onto Bogue Banks beaches has been limited, with most activity occurring, once again, on the easternmost and westernmost sections of the island.

### 2.3. Data Collection in the Field and Laboratory Processing

We report on numerous individual studies here. They were conducted under the auspices of multiple funding sources and with individual goals. However, all arose from our interest in answering the questions identified in the introduction. The specific relationships between the studies and the governing questions are indicated in Table 1. In addition, the majority of these separate projects utilized similar methods for collecting and quantifying fauna and sediments, which are described below, although exceptions to the general methods are identified as well in Table 1.

#### 2.3.1. Sediment Characteristics

Sediment samples were collected using either a handheld shovel or, most frequently, cores using a 2.5 cm diameter syringe to place ~150 g of sediment into pre-labelled Whirl-Pak bags or plastic containers. Upon returning to the laboratory, samples were held in a cold room at 7 °C until processing using procedures slightly modified from Folk [24]. Determination of grain sizes began when a sediment sample was transferred into 500 mL Erlenmeyer flasks to remove salt and organic matter from the samples. The flask was then filled to the top with deionized (DI) water, inverted several times to ensure complete mixing, and then allowed to sit, undisturbed for a minimum of 24 h. After resting, the supernatant was carefully decanted, without disturbing the sediments on the bottom of the flask. The sample was then flooded again, inverted, and allowed to rest for another 24 h. This washing procedure was repeated one more time before transferring the sediments to Al trays, which had been massed previously, and placed into a drying oven set at 80 °C. Periodically, each tray was removed from the oven and massed to the nearest 0.01 g using a top-loading Mettler balance. Samples were returned to the oven until they achieved constant mass. A dried sample was transferred to a stack of sieves (63, 125, 250, 500, 1000, and 2000 μm) that was then placed on a Ro- Tap and shaken for 20 min. Sediment retained on each sieve (and the bottom pan) was then massed to the nearest 0.01 g. Grain size metrics were calculated using Gradistat [25]. Data from historical studies (pre-nourishment, 1969–1970) employing granulometric analysis were made available for this project by the Institute of Zoology, University of Innsbruck (Rieger, unpublished—Table 1).

In 2006 we measured sediment penetrability in the lower intertidal of four beaches (Spinnaker’s Reach, Eastern Regional Public Access, Iron Steamer Pier, and Fort Macon). At each beach, penetration depth was determined by inserting a stiff, 2 mm diameter metal rod vertically into the sediments to the point of refusal. After noting where the sediment surface intersected the rod, it was removed and the length of the section that had been buried was measured to the nearest 1 mm. Three replicate insertions were made at each of 40 locations randomly located over 2 km along the beach. The mean depths of penetration at each location among the four beaches were compared using one-way analysis of variance (factor = beach).

#### 2.3.2. Meiofauna

Cores were collected using a 2.5 cm diameter syringe inserted vertically (5–6 cm deep) into the sediments for surface samples or, for samples at depth, by first digging a pit and then inserting the corer horizontally into the undisturbed side wall of the pit at specific depths. The contents of the corer were transferred immediately into pre-labelled 30 mL plastic centrifuge tubes that held 15 mL of a 10% formalin 32 μm-filtered seawater solution with Rose Bengal added. The tube was capped, shaken, and placed into a cooler. In the laboratory, the supernatant was pipetted off the undisturbed pellet in the bottom of each centrifuge tube and disposed of through a 63 μm mesh sieve. DI water was added to the centrifuge tube, the contents of the tube mixed by shaking, and, after the sediment, but not the organisms, settled to the bottom of the tube, the supernatant was decanted through the same sieve as used above. This mixing and decanting procedure was repeated two more times (10 preliminary trials where the remaining sediment was examined with dissecting microscope found that 95–97% of all fauna were removed from the sediments after three serial washings). All material retained on the sieve was washed into 5 mL centrifuge vials, flooded with 70% ethanol solution, capped, and stored until microscopic enumeration could be done. The contents of each vial were washed separately into either a gridded (1 × 1 mm) plastic tray or Bogorov counting chamber and examined systematically using Wild M5 dissecting microscopes. Fauna were identified to phylum or class level (Appendix A; Table A1) and enumerated.

#### 2.3.3. Identification and Enumeration of Species

From the late 1960s through to the mid-1970s, a series of studies were conducted on the Emerald Isle (EI) and Iron Steamer Pier (ISP) beaches to reveal meiofaunal diversity and document patterns of species distribution and behavior. Results from many of these studies were published [26–32] but some were not. Over the past several years we gained access to archived granulometric data sheets, species inventories, drawings of new species, wholemount slides, and resin-embedded meiofaunal extractions fixed for electron microscopy (Figure S1, [32]). The historical data available are highly detailed for turbellarians. Although most of the turbellarian species from these studies remain undescribed, a sufficient quantity of the archived information enabled us to match most of the historical helping-names to species currently collected (e.g., Table S1). Details of contemporary methods for examination, preservation, and a family-level key to identification of turbellarian specimens can be found in Smith et al. [33]. We use “turbellaria” not as a formal taxon but as a collective name for taxa within the Acoelomorpha and Platyhelminthes [34].

### 2.4. Metabarcoding: Bogue Banks versus Bear Island

To maximize the contrast between a disturbed, nourished exposed beach and a relatively pristine exposed beach we collected samples for metabarcoding from ISP and BI. ISP is a tourist beach with extensive (condominium) beachfront development that is served by a public parking lot and nourishment has occurred there in 2002, 2007 and early 2013. BI can only be reached by boat (resulting in limited tourist visitation), does not have any residential development, and has only received one small dredge disposal (~5000 m^3^ in 2009) that had no measurable effects on grain size characteristics or macrofaunal abundances after 6 months [35].

On the 28th of May 2017 (ISP) and the 10th of June 2017 (BI), sediment samples were collected at six tidal heights (mean high water—Sta I, intermediate between mean high water and mean tidal level—Sta II, mean tidal level—Sta III, intermediate between mid-tidal level and mean low water—Sta IV, mean low water—Sta V, and swash below MLW—Sta VI). Sediment, from depths depending on tidal height (Sta I, 10–50 cm; Sta II, 0–40 cm; Sta III, 0–30 cm; Sta IV, 0–40 or 0–45 cm; Sta V, 0–20 cm, Sta VI, 0–10 cm), was placed into separate, triplicate, sterile 50 mL conicals. Each conical was immediately frozen on dry ice. In the laboratory sediment was thawed, re-suspended, and organisms elutriated in 1 L of ice-cold sterile Instant Ocean™. Organisms were captured on a 32 μm sieve and concentrated by centrifugation. Organisms were transferred to a bead-beating tube (0.5 mm glass beads), bead-beaten for 1 min in Qiagen ATL buffer with added proteinase K, and otherwise treated according to our standard DNeasy protocol [36]. After elutriation, sediment was rinsed 3× in tap water, dried at 80 °C overnight, and analyzed as described above.

For next-generation sequencing, DNA samples were sent to HudsonAlpha where amplicons were generated using primers targeting V4/V5 of the 18S rDNA gene (modified from [37]). HudsonAlpha prepared libraries and sequenced them (MiSeq, 300 bp, PE), returning demultiplexed reads trimmed of barcodes, adapters, and forward primers. QIIME was used for read-joining, quality- filtering, and trimming of reverse primers [38]. QIIME (pick_open_reference_otus.py, 99%), and Usearch -cluster_otus (97%), and -unoise3 (100%) as implemented in Usearch64 (V.10) were used for OTU clustering and OTU table-generation (-otutabout). Taxonomy assignment (RDP classifier against the Silva132 database) and core diversity analyses were conducted in QIIME as well.

### 2.5. Sediment Selection Experiment

**Figure 2.**
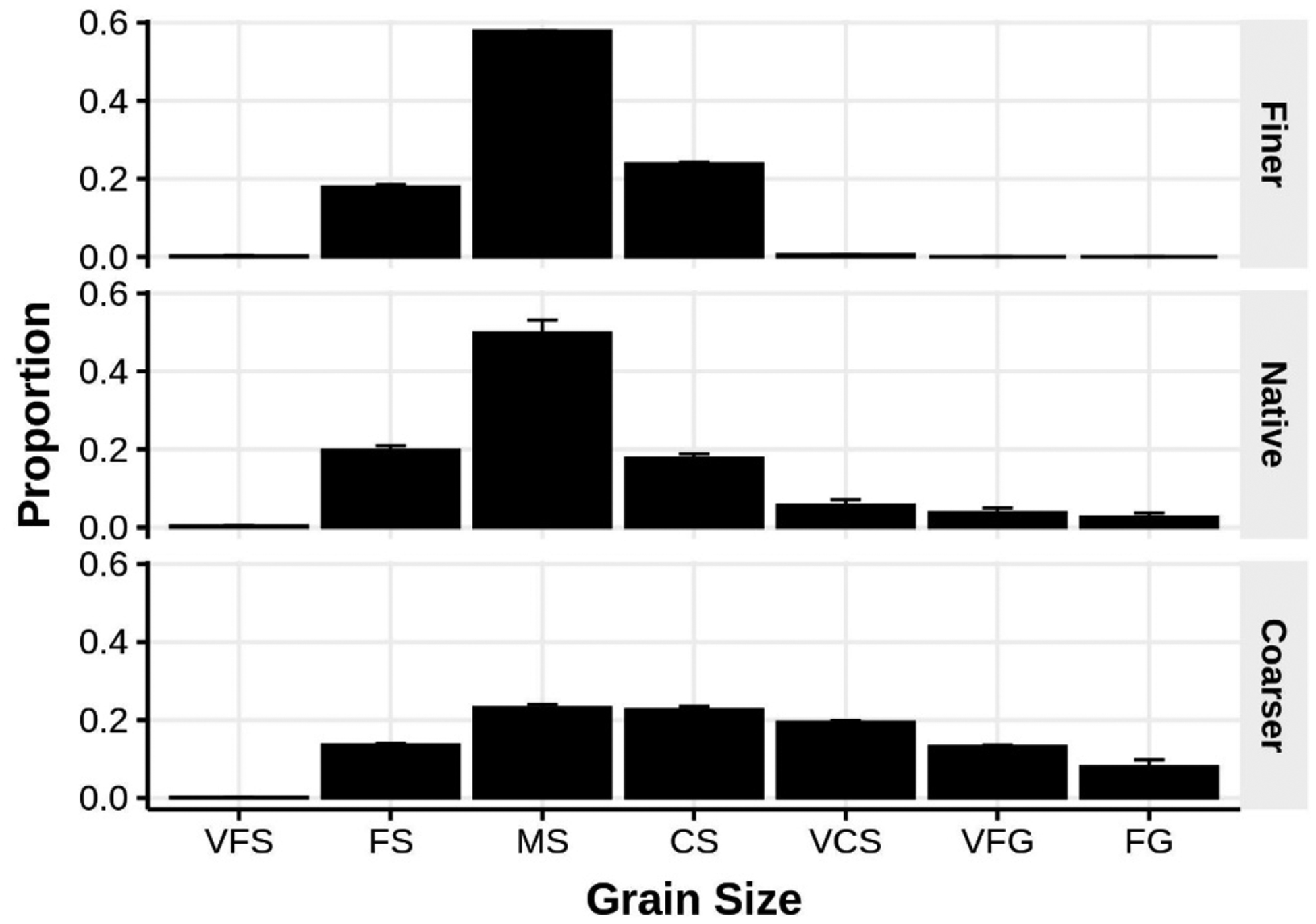
Comparison of the mean (±1 SE) grain size distributions of sediments used in the experimental cage study (unmanipulated, native sediments are in the central panel). Sediment fraction codes: VFS = very fine sand, FS = fine sand, MS = medium sand, CS = coarse sand, VCS = very coarse sand, VFG = very fine gravel, and FG = fine gravel.

To determine whether exposed beach meiofaunal assemblages colonize coarser and finer sediments differently, we deployed 16 meshed cylinders in the field containing sediment crafted into one of two different granulometry compositions. The sediment combinations placed into the cylinders were derived from sediment samples taken from the same beach and tidal height where we planned to conduct the experiment. After initial collection, the sediments were washed repeatedly with DI water to remove all organisms and salt, dried in an oven set at 80 °C to constant mass, and separated into 1 phi fractions using a Ro-tap. The two sediment combinations intended for the experimental cylinders were created by recombining the sorted sediment fractions selectively to produce two distinct, but homogeneous, combinations: one finer and one coarser than native sediment typically observed at the study site (Figure 2). The finer sediment was well-sorted and dominated by medium sand while the coarser fraction was poorly-sorted with almost equal proportions of medium, coarse, and very coarse sands.

Each experimental unit consisted of a 5 cm long, 5 cm diameter polypropylene cylinder. One end of each cylinder was covered with 1 mm mesh held tightly in place with a plastic zip tie. We completely filled each cylinder with either finer or coarser sediment that had been slightly dampened with DI water to maintain coherence of the sediment in the cylinder. Once filled, the open end of each cylinder was covered with 1 mm mesh held in place with a zip tie. Each filled and meshed cylinder was tightly wrapped in plastic to prevent loss of moisture or grains through the mesh during transportation to the beach.

On 18 July 2016, at ISP, we dug a 2.5 m long, 15 cm wide, along shore-parallel trench to a depth of 35 cm at MLWN using shovels. At six haphazardly-selected locations on the inside of the trench, within 3 cm of the bottom of the trench, we collected a core of undisturbed sediment using a 2.54 cm-diameter syringe corer inserted horizontally into the trench wall. The contents of each core sample were placed immediately into a pre-labelled, 50 mL centrifuge tube, which was capped and placed in a cooler. We then placed the sediment-filled cylinders into the trench. Cylinders with finer and coarser sediments were deployed together in pairs, in contact side by side, on the bottom of the trench, with the meshed ends facing towards the ocean and upper beach. The distance between each pair of finer and coarser sediment cylinders was approximately 25 cm. Prior to filling the trench with the excavated beach sediment, a 2-m long rule was placed on top all eight pairs of cylinders to aid in relocation. The location of both ends of the trench were determined to the nearest cm using an RTK GPS.

In the laboratory, each centrifuge tube was filled with a sufficient volume of 10% formalin, with added Rose Bengal, to cover the sediment. The tube was then capped, shaken, and left undisturbed for 48 h. The supernatant formalin in each tube was decanted through a 63 μm sieve. The sample was then washed four more times with deionized water with mixing and subsequent decantation of the supernatant through the same sieve. All material retained on the sieve was then washed into a pre-labelled, 2.5 mL centrifuge tube that was then filled with 50% ethanol and capped. Enumeration of the major taxa of meiofauna was made by pipetting each sample into a Bogorov plankton counting dish and examining with a Wild M5 dissecting scope

On 22 July 2016, we returned to the beach, relocated the eastern end of the trench using the GPS and carefully excavated sediment by hand until we found the easternmost pair of cylinders and the overlying plastic rule. Continuing to excavate by hand, we followed the rule to expose the other seven cylinder pairs. The paired cylinders were separated and each cylinder was placed into a pre-labelled plastic bag after removing excess sediment from the outside of each cylinder using a brush. The tightly bagged cylinders were then placed into a cooler. In the laboratory, the contents of each cylinder were washed into 1-L plastic jars using filtered (32 μm) seawater. A volume of 10% formalin, with Rose Bengal, equal to approximately twice the apparent volume of the sediments in each jar was added and the sample was mixed by shaking after the jar was capped. After 48 h the meiofauna in these samples were retrieved and enumerated using the same procedure as used for the initial core samples, except for the use of the 1 L jars instead of the 50 mL centrifuge tubes.

### 2.6. Short-Term Responses of Meiofauna to Beach Sediment Disposal

We used a moderate-scale (~700,000 m^3^) beach disposal project targeted for a Pine Knoll Shores beach to test for short-term (days to weeks) effects of nourishing on meiofaunal abundances in the low intertidal. On 17 January 2008, one week prior to the expected beginning of sediment disposal, we collected meiofaunal and sediment samples at six sampling locations: two sites that would occur within the beach area to be nourished, two sites (one east and one west) outside the planned nourishment area but within 0.5 km, and two sites (one east and one west) outside the nourishment area by at least 3 km. On our first sampling day we collected, just above the swash zone (~MLW), a surface sediment core and three replicate meiofaunal cores using the same procedures described above (2.3.1 and 2.3.2) from three blocks separated by at least 3 m. The samples were processed in the laboratory as described above. Disposal began on 21 January 2008. We visited each site and sampled in the same manner on three subsequent occasions: 12 February 2008 (during nourishment); 20 March 2008 (immediately after nourishment activities ended); and 30 April 2008 (approximately 5 weeks after nourishment ceased).

### 2.7. Statistical Analyses

As we were not testing explicit hypotheses, the specific analyses we employed were selected to reveal patterns and to test whether those patterns differed from random. Except where identified below, in most studies the replication was relatively low (*n* = 5) and variation among samples relatively high. Under these conditions meeting the assumptions of analysis of variance can be challenging, so we preferentially used nonparametric tests. For paired comparisons we used Mann–Whitney comparisons, for one-way designs we used Kruskal–Wallis tests, and two-way designs we used Scheirer–Ray–Hare tests [39]. All univariate tests were conducted in R [40] employing the following packages: *FSA* [41], *ggpubr*, and *rcompanion*. Where replication of samples was adequate and parametric assumptions were met by the data, ANOVA and linear regression were used (instances described below).

Testing for significant differences in multivariate assemblages was accomplished using permutational analysis of variance (PERMANOVA, 999 iterations) conducted in the R package *vegan* [42]. Tests for differences of both centroids and dispersion of multidimensional arrays were examined in each case. Using the same package, analysis of similarity percentages (SIMPER) was used to determine which taxa contributed significantly to significant PERMANOVA results and what percentage of dissimilarity each taxon contributed. Visualization of ordination results were made using non-metric dimensional scaling (nMDS) utilizing Bray–Curtis dissimilarities calculated from square-root transformed abundances. In each case, we overlay the taxa onto the nMDS plot to indicate associations of each taxon to each other and to the plotted samples.

## 3. Results

### 3.1. Sediment Characteristics

#### 3.1.1. Comparisons across Nourishment Eras—General Question #1 (In Part)

**Figure 3.**
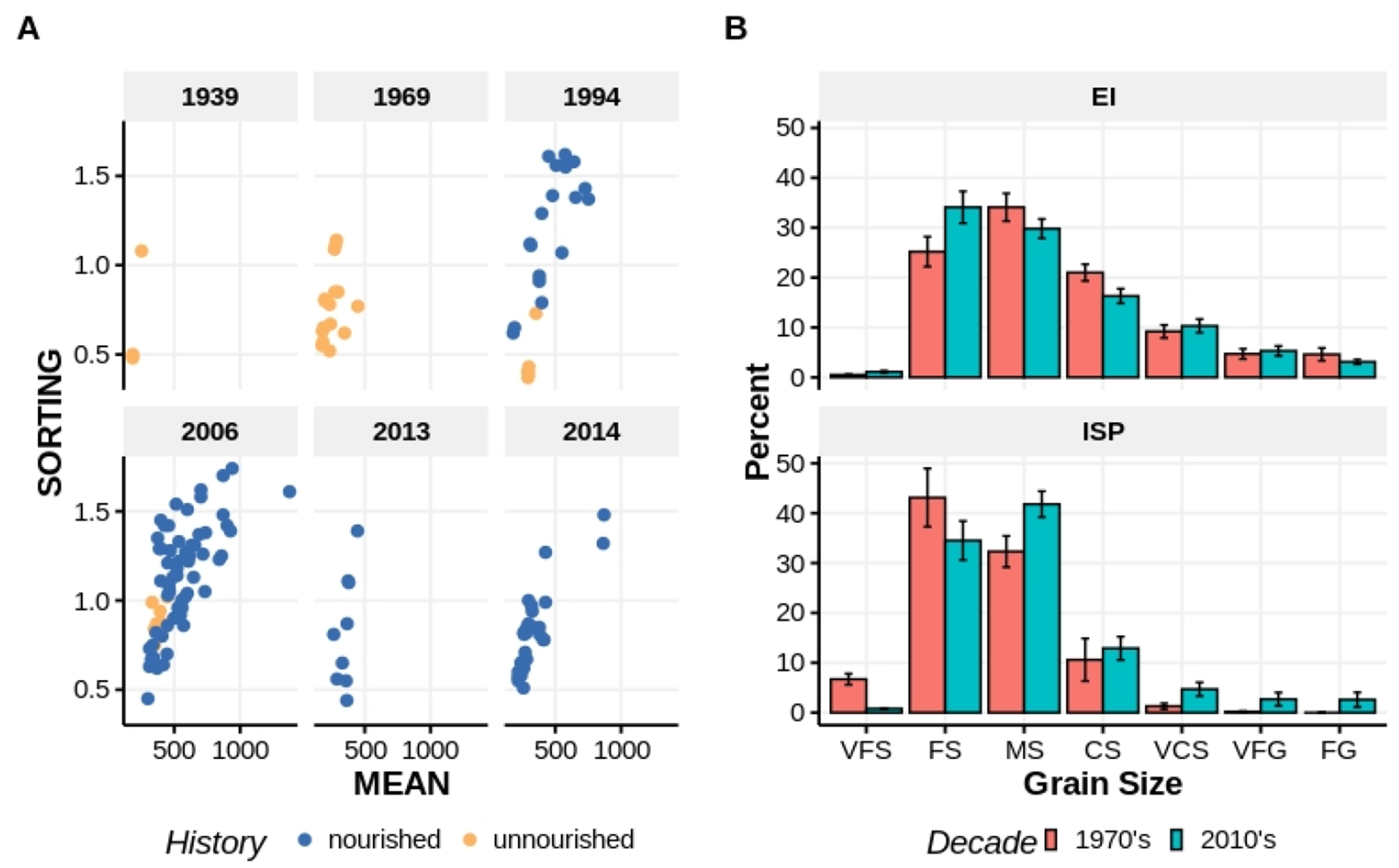
Comparisons of mean grain size (μm) versus sorting (φ) from the beach surface (0–10 cm) reported in separate studies spanning >60 years (**A**). Teal samples were taken from beach areas that had never been nourished. Red samples were taken from beach areas that had been nourished from <1 to >10 years prior. Percent composition of sediment fractions for samples taken at EI and ISP in the 1970s and again, at each beach, in the 2010s (**B**). Sediment fraction codes are the same as in Figure 2.

We compared sediment mean grain size and sorting garnered from Bogue Banks historical studies (1939—[43]; 1969—[28]; 1994—[44]) to grain median and sorting statistics derived from our samples (Figure 3). Pre-nourished Bogue Banks beaches lacked the coarser and poorly-sorted sediments that we observed routinely in the past two decades. Locations with fine, well-sorted sediments remain into the 2000s, but coarser, poorly-sorted surface sediment is a persistent contemporary feature of Bogue Banks beaches, even years removed from discrete nourishing events as indicated by samples from 2013 to 2014. Comparing samples taken from these last two years to those taken in 1994 and 2006, which were within a year to a decade of nourishing, suggests that tidal and wave reworking of the beach has fined the surface sediments, although sorting remains poor generally.

Comparing Rieger’s raw sediment data collected from EI and ISP in the 1970s to sediment data we collected from the same beaches and tidal heights in the 2010s revealed that sediment character at EI did not differ significantly across decades, but that ISP did differ significantly over the same time interval (Figure 3). In the 2010s, ISP sediments were coarser (mean (±1 SE) 1970’s median = 2.23 (0.18) φ vs. 2010s median = 1.79 (0.15) φ, Mann–Whitney *p* = 0.019) and more poorly sorted (mean (±1 SE) 1970’s sorting = 0.75 (0.04) φ vs. 2010’s median = 1.00 (0.10) φ, ANOVA *p* = 0.025).

**Figure 4.**
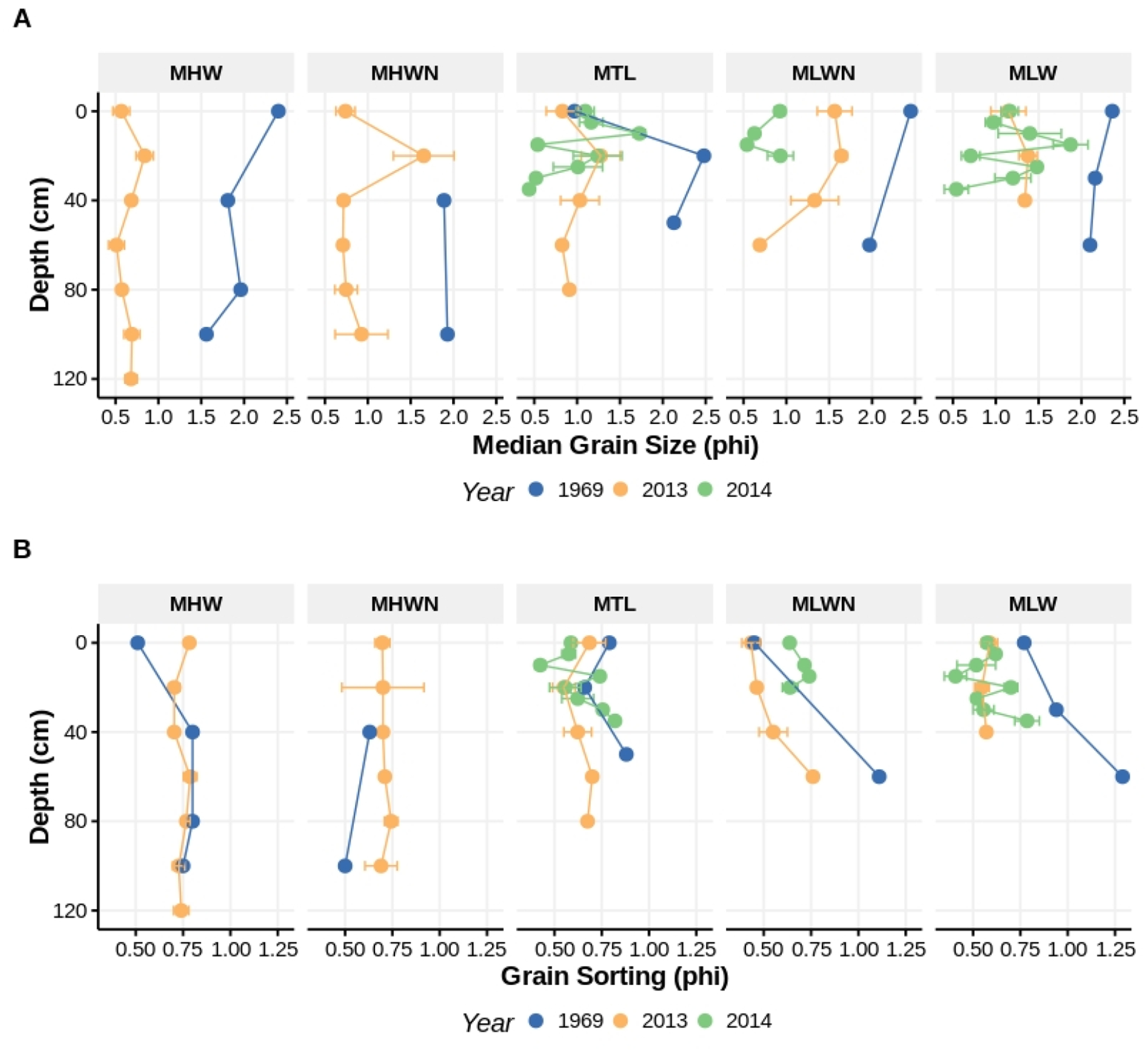
Comparison of median grain size (**A**) and sorting (**B**), both measured in phi, from the beach surface to depth at several intertidal heights at Iron Steamer Pier Beach. The historical data (blue) are from Lindgren (1972). The 2013 and 2014 (yellow and green, respectively) data, collected from the same location as the Lindgren data, are means and have standard errors associated with them. Tidal heights: MHW = mean high water; MHWN = mean high water neap; MTL = mean tidal level, MLWN = mean low water neap; and MLW = mean low water.

Examination of sediment composition with depth indicates that coarser, poorly-sorted sediments remain at ISP even ~20 years after nourishment (Figure 4). In 1969, sediments coarsened moderately or stayed the same with depth (except at the surface at MSL). From MTL to MLW sediments were less well-sorted with depth, while sorting changed little with depth in the higher intertidal. In 2013 and 2014, at the same location as in 1969, sediments were much coarser at all tidal heights and at almost all depths. Sorting with recent sediment samples are not that different than in 1969. The fining of surface beach sediments since nourishment occurred suggested by the results described in the preceding paragraph appears limited to the surface and is not a feature of the beach at depth: coarse, poorly-sorted sediments, introduced during nourishment, persist in all of these beaches below the sediment surface.

#### 3.1.2. Contemporary Comparisons among Beaches—General Question #2 (In Part)

**Figure 5.**
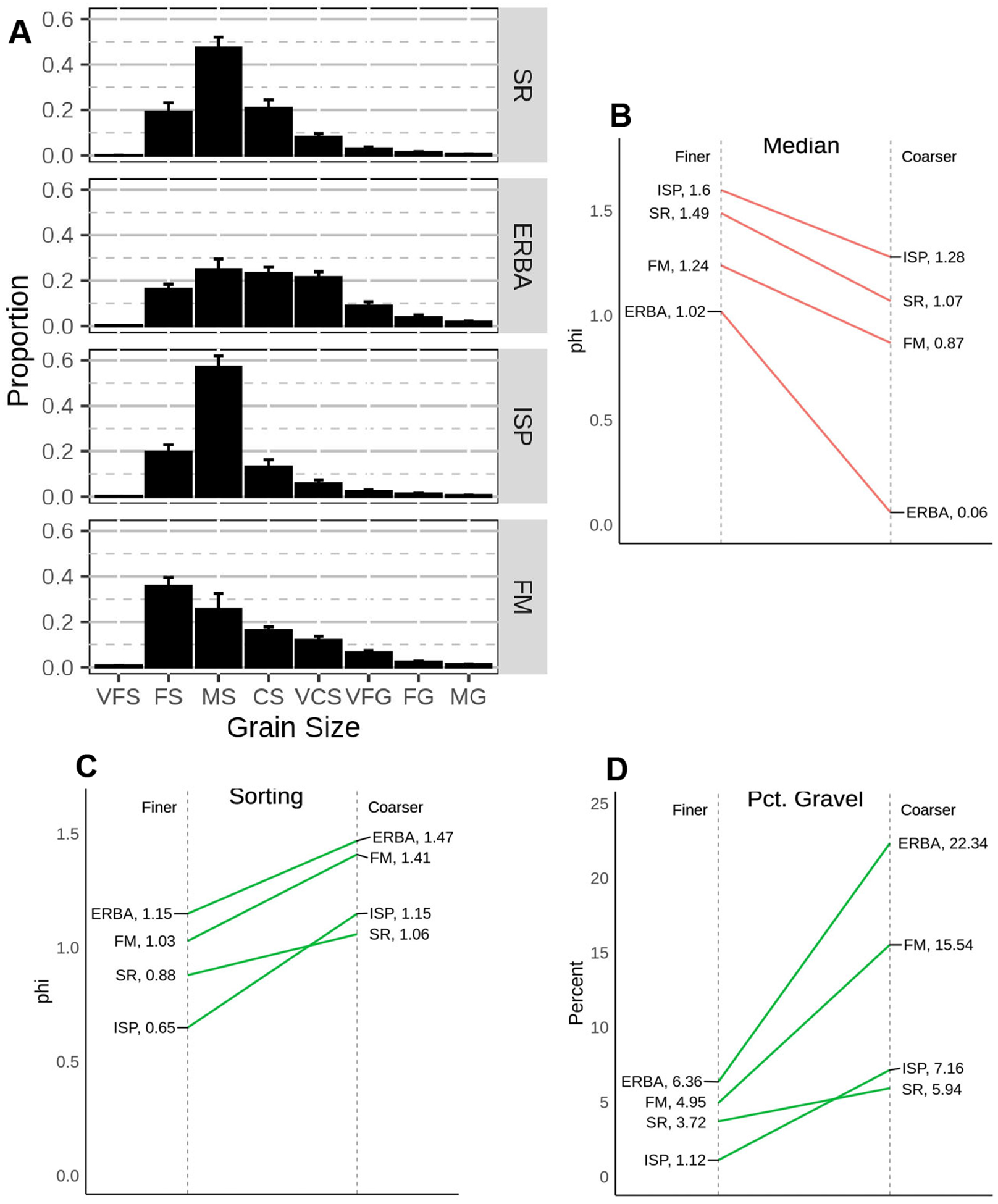
Relative grain size distributions of sediments collected in the low intertidal of several Bogue Banks beaches in June of 2006: (**A**) beach codes same as in Figure 1, grain size category codes same as in Figure 2. Panels (**B**–**D**) illustrate some sediment parameters for the “finer” and “coarser” samples collected from each beach. Green lines indicate that the respective value is lower in “finer” sediment samples, red lines indicate higher values in the “finer” sediment samples.

A comparison of surface sediment composition from Bogue Banks beaches in 2006 demonstrates how much sediment composition varies in locations with different nourishment histories. In 2006 Spinnaker’s Reach (SR) had not yet experienced direct placement of any sediment from nourishment. Some nourishment sediments could have drifted to that location as the prevailing net longshore drift is east to west and up-current locations had been nourished prior to our sampling. SR was dominated by medium sands, with almost equal contributions of fine and coarse sand with very little gravel (Figure 5). On the opposite east end of the island, at Fort Macon (FM), beach sediments were dominated by fine sands (most of the spoil placed on multiple occasions in this location is dredged from a nearby inlet and port harbor) with moderately more gravel than observed at SR. Eastern Regional Beach Access (ERBA) sediments were coarse with the highest proportion of gravel seen at any location and with almost equal contributions of very coarse and coarse sands as medium sand. ISP had about the same amount of gravel as Spinnaker’s Reach beach and more medium sand (>60%) than any of the other beaches. These differences in composition demonstrate that: 1) beaches within a few km of each other can vary considerably in composition and 2) interstitial environments, which depend on the proportions and mixing of fine and coarse sediment particles, likely differ as well.

**Figure 6.**
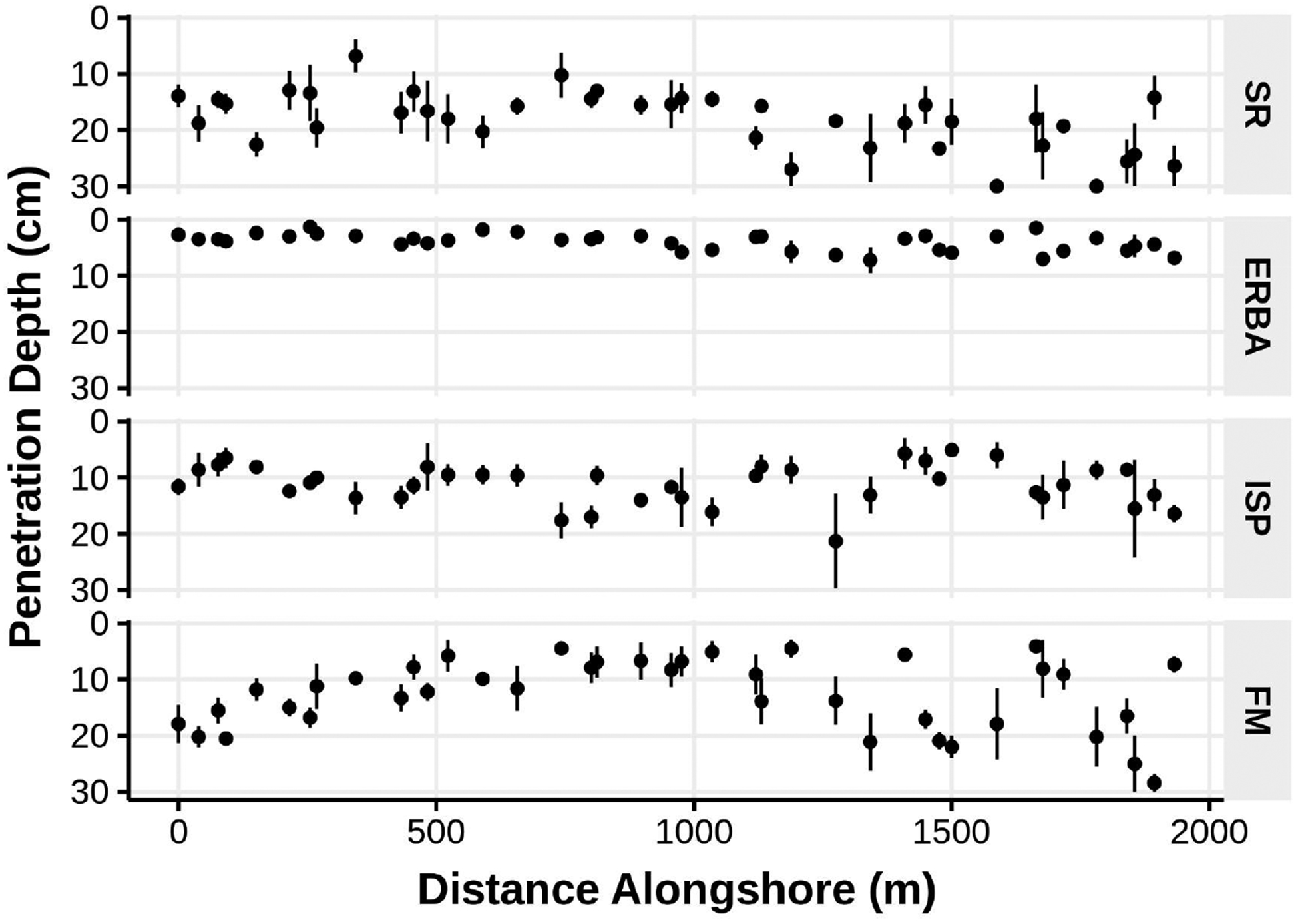
Mean (±1 standard error) vertical sediment penetrability of low intertidal sediments at random intervals alongshore several Bogue Banks beaches in June of 2006 (beach codes same as in Figure 1).

Sediment penetrability provides an indication of how differences in sediment composition can affect beach characteristics important for fauna. There were significant (one-way ANOVA *p* < 0.002) differences in penetrability over alongshore distances of hundreds of meters among the beaches with the shallowest penetrable mean depth at ERBA and the deepest at SR (Figure 6). The ability of shore birds to probe for food, for burrowing macrofauna to enter the sediments, and for water to percolate (and presumably, for meiofauna to move) would differ among these beaches.

### 3.2. Meiofauna Historical Comparisons—General Question #1 (In Part)

Across all studies reported here, nematodes, copepods, gastrotrichs, and turbellarians were omnipresent and numerically dominant compared to other taxa. We also found ostracods, tardigrades, mystacocarids, archiannelids, and ciliates but these taxa were sporadic in appearance and very uncommon even when found. With few exceptions, identified below, analysis of abundances and presence/absence patterns of minor taxa provided no additional information or insight and are not presented.

#### 3.2.1. 1969 versus 2013

**Figure 7.**
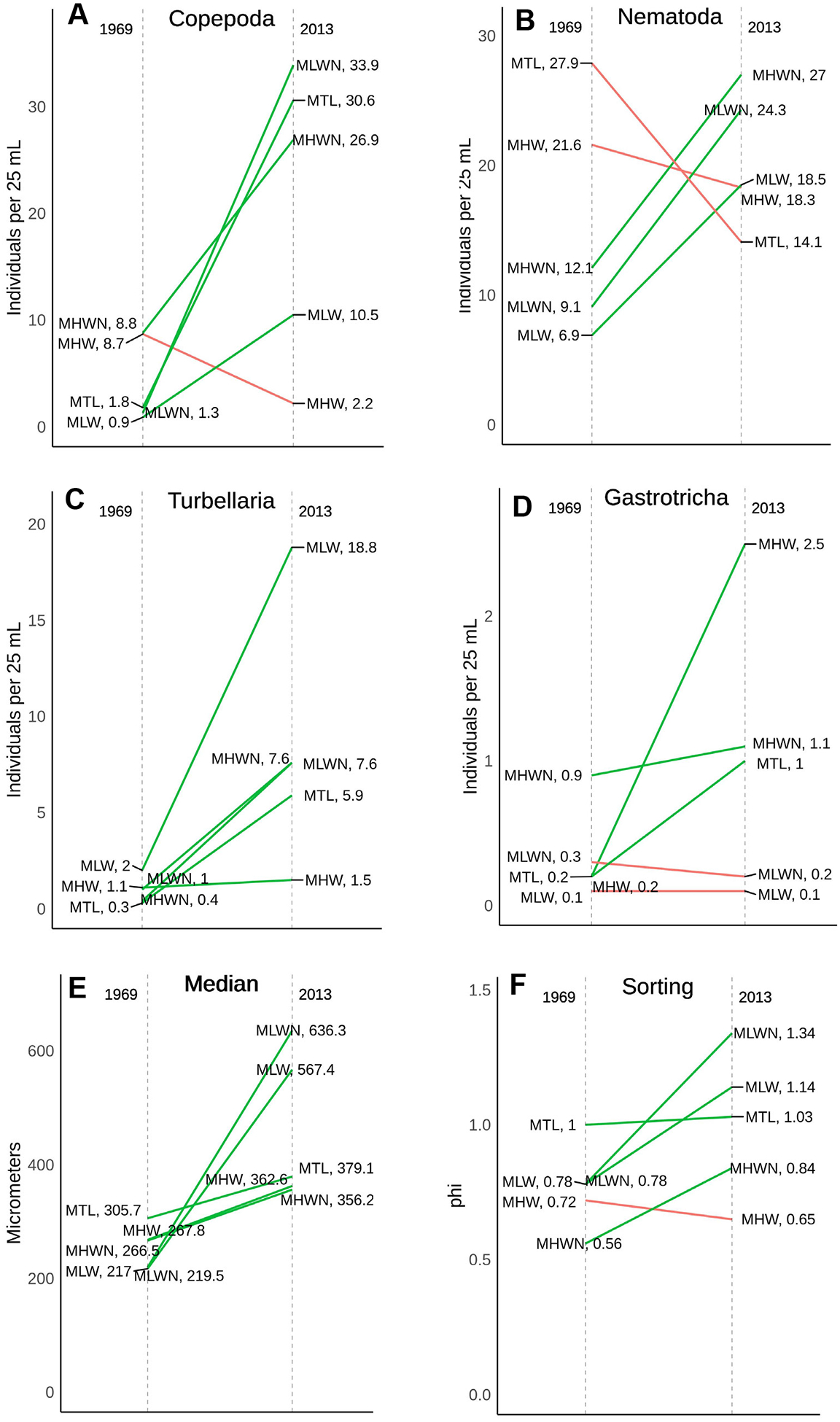
Historical comparison of the more abundant meiofaunal taxa (**A**–**D**) and grain size parameters (**E,F**) from samples collected in the same Iron Steamer Pier beach locations (tide and depth) in 1969 and 2013. Colors and tidal height abbreviations as in Figure 5.

As mentioned above, ISP sediments in 1969 were finer at all tidal heights compared to 2013 (Figures 4 and 7). Generally, absolute abundances of each major taxon were higher at each tidal height in 2013 than 1969 except for copepods at MHW, nematodes at MHW and MSL, turbellarians at MHW, and gastrotrichs at MLWN and MLW (Figure 7). Apart from gastrotrichs, all between-year comparisons were significant or nearly so (Table 2). Turbellaria and gastrotrichs showed significant differences among tidal heights, but not for the interaction of year × tidal heights. A significant interaction of year and tidal heights occurred only for copepods where post-hoc comparison revealed that copepods were far more abundant in the middle of the beach (MHWN, MTL, and MLWN) in 2013 than in 1969 (MHW and MLW were not significantly different across eras).

**Figure 8.**
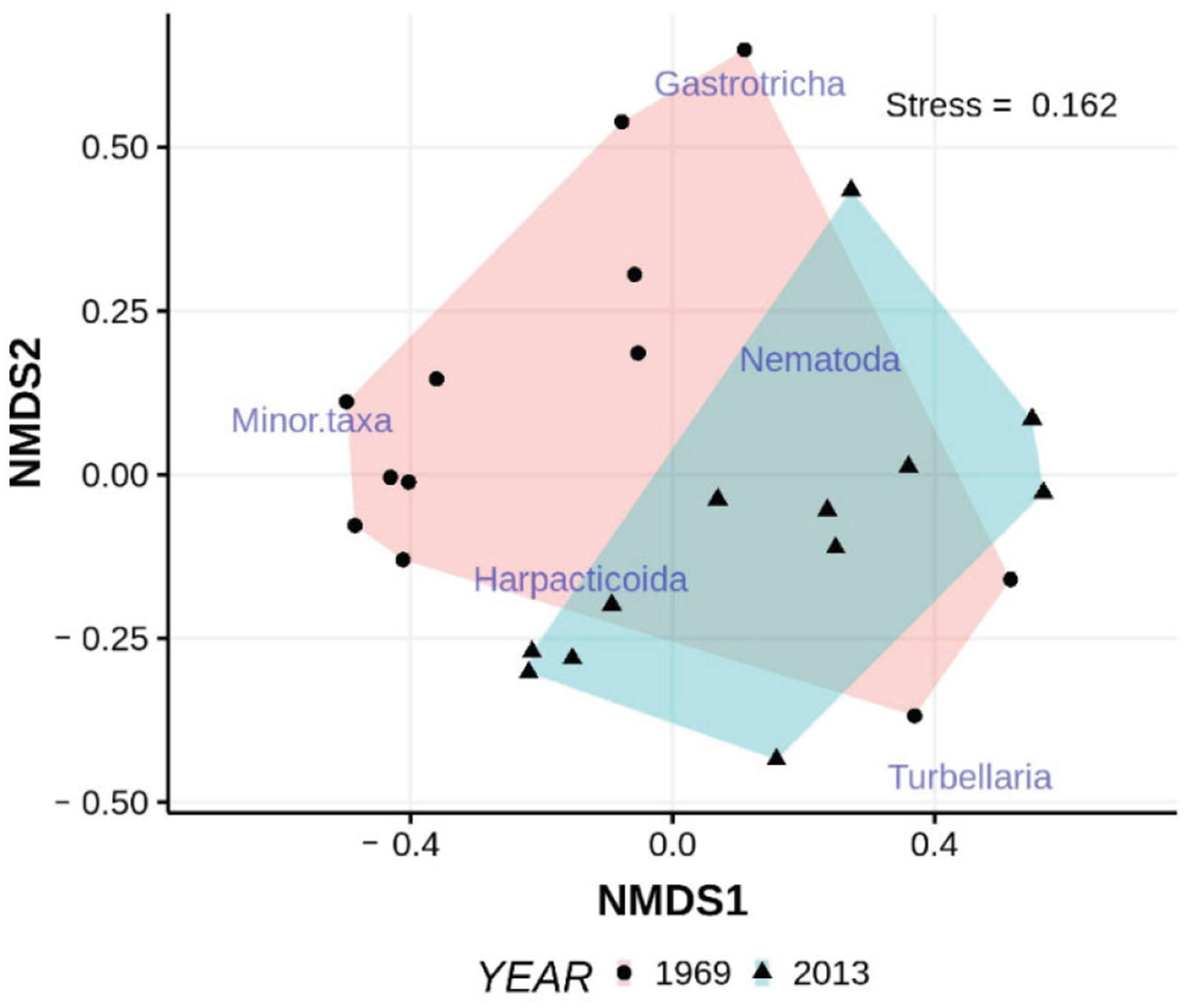
nMDS ordination of taxon abundances collected in samples from ISP beach intertidal in 1969 and 2013. “Minor” taxa here include tardigrades, archiannelids, juvenile bivalves, and juvenile polychaetes, all of which only occurred infrequently in low numbers. Non-metric scales serve to ease visual estimation of dissimilarity distances but are not associated with any specific units.

Multidimensional analyses indicated that the meiofaunal assemblages (consisting of each major taxon plus counts of low-abundance taxa such as tardigrades and ostracods pooled into minor taxa), differed across eras (Figure 8). PERMANOVA indicated a significant difference (*p* < 0.004, homogeneity of multivariate dispersions *p* > 0.143) between years. SIMPER indicated that nematodes (39%) and harpacticoids (33.5%) contributed significantly to the dissimilarity between years. Examination of the vectors associated with the nMDS ordination indicate that grain sorting contributes more to differences in faunal structure than median grain size.

#### 3.2.2. 1970/1976 versus 2012

##### Major Taxa

**Figure 9.**
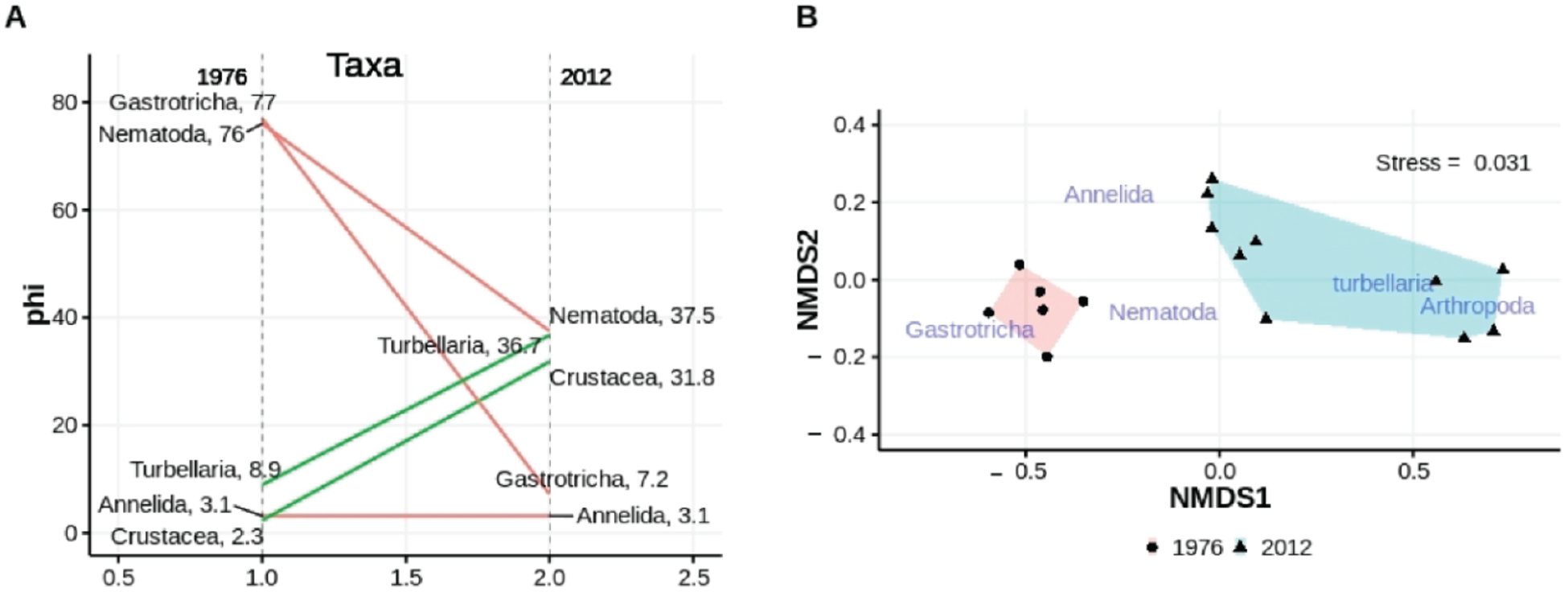
Univariate (**A**) and multivariate (**B**) comparisons of abundances of major taxa collected from the same tidal height at ISP in March 1976 and March 2012; non-metric scales serve to ease visual estimation of dissimilarity distances but are not associated with any specific units.

Separate Mann–Whitney tests comparing abundances of major taxa collected in the low intertidal of Emerald Isle (EI) in March 1976 to those observed at the same beach at the same tidal height in March 2012 reveal that gastrotrich abundance was 10× lower (*p* < 0.009) by the later date while crustaceans (mostly harpacticoid copepods but the Mystacocarid *Derocheilocaris* as well) and turbellaria were significantly (*p* < 0.0003 and *p* < 0.0001, respectively) more abundant over the same time interval (Figure 9). Nematode abundances decreased but the change was not significant (*p* = 0.356). Annelids (*Diurodrilus* and *Microphthalmus*) showed no change in abundances (*p* = 0.737). The differences in abundances were reflected in multivariate results as well (Figure 9). PERMANOVA indicated a significant difference (*p* < 0.001, homogeneity of multivariate dispersions *p* > 0.213) between years. SIMPER indicated that gastrotrichs (61.6%) and nematodes (17.5%) contributed significantly to the dissimilarity between years.

##### Turbellarian Species Diversity—General Question #4

In early 1970, Rieger sampled the ISP site and identified 28 species of turbellarians (2634 specimens), assigning them helping-names (unpublished but original data were available to us). Our studies over the last 20 years have identified all but two of these species, and, unsurprisingly, uncovered additional species, some observed by Rieger at other sites on Bogue Banks, and some, unobserved by Rieger (Table 3). Similarly, we can compare Rieger’s data from an unpublished survey made at EI (August 1970) to our contemporary results. Rieger identified approximately 81 species of turbellaria and gave them helping-names, using 350 samples covering the entire intertidal and shallow subtidal (Table S1). Our work at the EI site has identified and sampled morphospecies for all but 19 of the species that Rieger found. We have placed particular emphasis on Kalyptorhynchia, and it appears that neither *Eukalyptospirale* nor *Schizocarcharodo* now occurs at EI. In addition, we have identified 25 species that regularly occur at EI that were not found by Rieger in his one-month sampling effort. The EI site is close to Bogue inlet (<1 km historically; currently ~2 km). We hypothesize that some of the differences between historical and contemporary species lists arise from inlet (or subtidal) species that temporarily settle out of suspension at high enough densities to be recognized.

### 3.3. Contemporary Comparisons of Meiofaunal Community Structure between Coarser and Finer Sediments

#### 3.3.1. Alongshore Island Patterns—General Question #2 and #3 (In Part)

**Figure 10.**
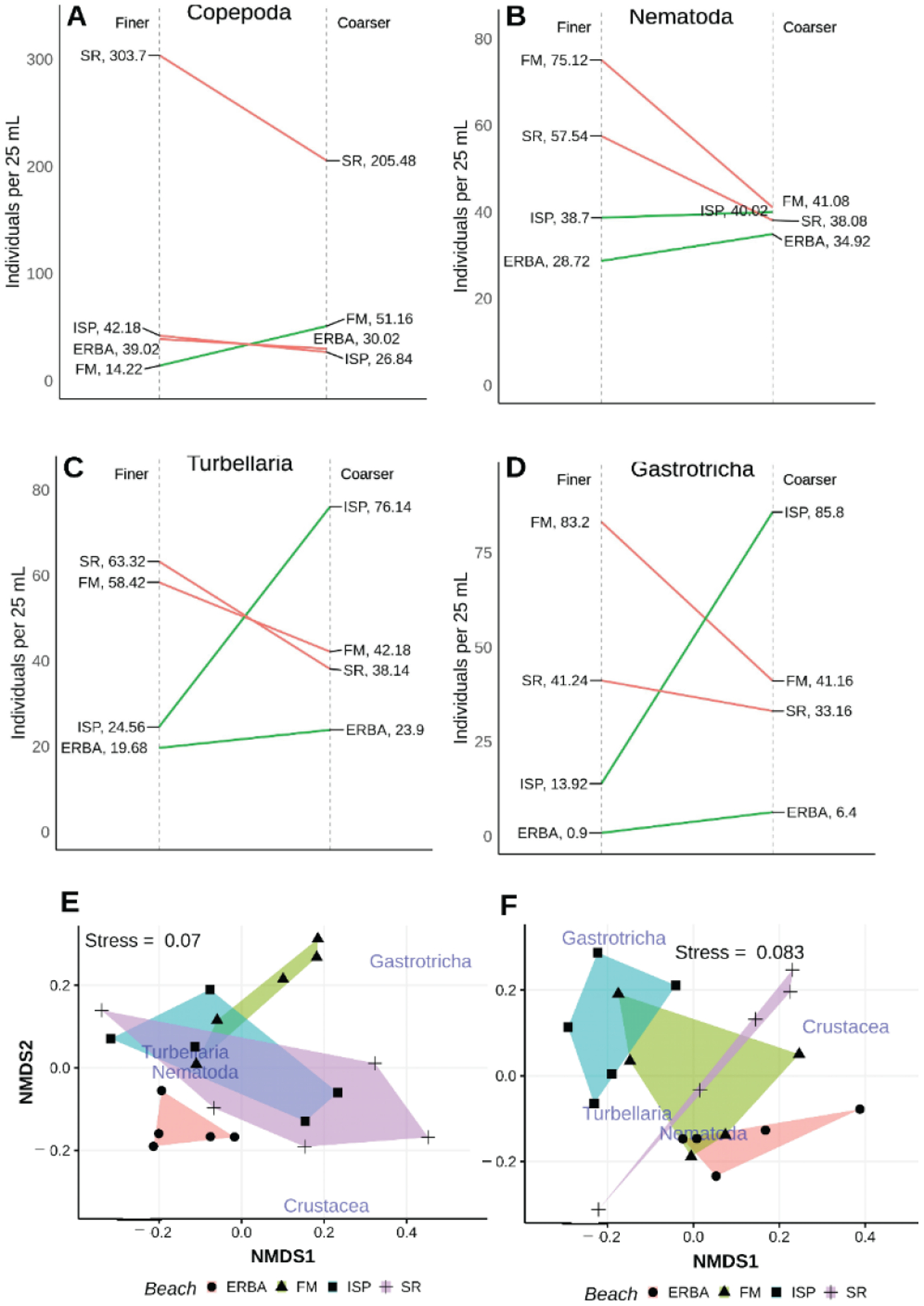
Univariate (**A**–**D**) comparisons of mean taxon abundances collected in low intertidal, surface finer and coarser sediment samples from four different beaches in 2006. Finer (**E**) and coarser (**F**) multivariate results are disaggregated to ease visual comparison; non-metric scales serve to ease visual estimation of dissimilarity distances but are not associated with any specific units.

In 2006, the association of different taxa with finer or coarser surface sediments varied depending on the beach that was sampled (Figure 10). Grain size analysis indicated that our visual ability to select finer or coarser substrate within a beach was effective. At all beaches median grain size, sorting, and percent gravel are all higher for coarser samples (Table 4, Figure 5). However, across beaches there was overlap of finer and coarser sediments. For example, the median grain size for sandy sediments at ERBA is intermediate between median grain sizes for coarser sediments seen at FM and SR. Given how variable and overlapping sediment parameters are among beaches, it is not surprising that faunal patterns were not consistent across beaches (Figure 10). Copepods were highest in abundance among all beaches at SR, but only showed a significant decrease in abundance in coarser sediments at that beach. Gastrotrichs were most abundant in coarser sediments at ISP and in finer sediments at FM. In both cases, the differences in abundance between coarser and finer sediments were significantly different in those two beaches. Nematode abundances were nonsignificant across all beaches and sediments types. Turbellaria were least abundant in both sediment types at the beach that had the overall coarsest substrate, ERBA. The only significant difference among turbellarian abundances in finer versus coarser sediments occurred at ISP.

The variation in patterns of individual taxa among beaches produced significantly different assemblages at the different beaches (PERMANOVA beach × sediment type *p* < 0.02, dispersion *p* = 0.31; Figure 10). The most distinct assemblages were those associated with shelly sediments at ISP, dominated by gastrotrichs (% dissimilarities 39–43%) and turbellaria (% dissimilarities 28–32%), and those in both sandy and shelly sediments at EI where gastrotrichs were almost absent and crustacea dominated (% dissimilarities 60–68%). Nematode dissimilarities (~30%) contributed to significant differences in few instances.

#### 3.3.2. Major Taxa Association (At Surface and at Depth)—General Question #3

**Figure 11.**
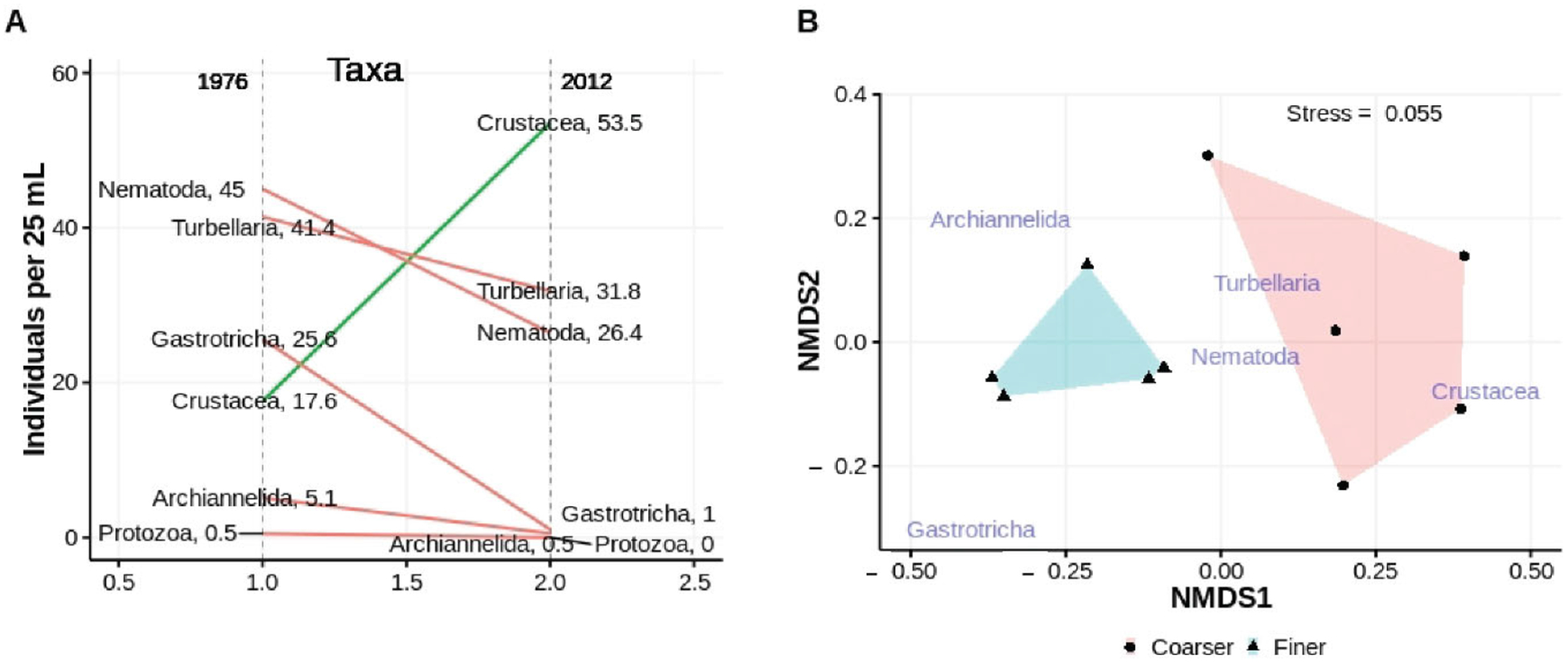
Univariate (**A**) and multivariate (**B**) comparisons of mean taxon abundances collected in low intertidal, surface finer and coarser sediment samples from ISP in 2012; non-metric scales serve to ease visual estimation of dissimilarity distances but are not associated with any specific units.

Both univariate and multivariate analyses indicate that fauna associated with finer and coarser surface sediments differed in the mid to low intertidal of EI beach when sampled in March of 2012. Nematodes and gastrotrichs were significantly less abundant in coarser sediments (Figure 11, Table 5). Copepods, although 3× more abundant in the coarser sediment samples, did not differ significantly between sediment type. Turbellaria also did not differ between sediments of different character. The multidimensional analyses indicated that the meiofaunal assemblages differed across sediment types (Figure 11). PERMANOVA indicated a significant difference (*p* < 0.01, homogeneity of multivariate dispersions *p* > 0.143) between sediment types. SIMPER indicated that copepods (50%) and gastrotrichs (20.1%) contributed significantly to the dissimilarity in assemblages associated with finer and coarser sediments, although only the latter were significantly different in univariate analyses.

**Figure 12.**
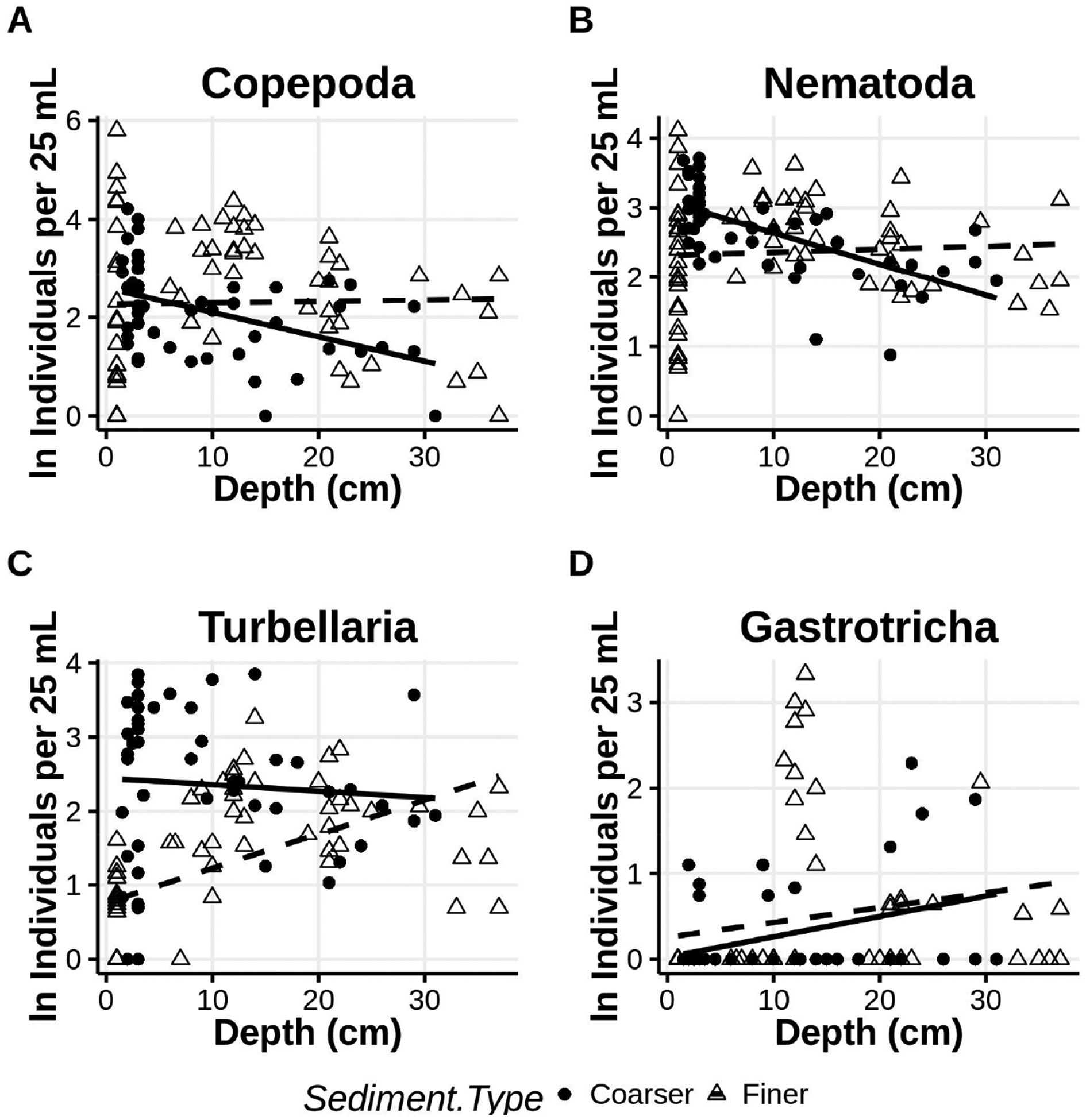
Abundances of major taxa **(A**–**D)** associated with finer and coarser sediments at depth into the beach from the sediment surface. Regression lines of ln abundance onto depth are partitioned by sediment type, coarser = solid, finer = dashed.

Abundances of several major taxa changed with increasing depth in the sediments in the low intertidal of ISP depending on whether the stratum of sediments at a given depth was finer or coarser (Figure 12). For nematodes and copepods abundances were largely constant with depth in finer sediments but decreased significantly if the deeper sediments were coarser (Table 6). In contrast, turbellaria increased in abundance with depth if the sediments were finer, but showed no significant difference if the sediments were coarser. Gastrotrichs were not abundant enough throughout the top 35 cm of beach sediment to reveal a significant depth relationship. However, the majority of all individuals collected were from a single stratum of finer sediments.

**Figure 13.**
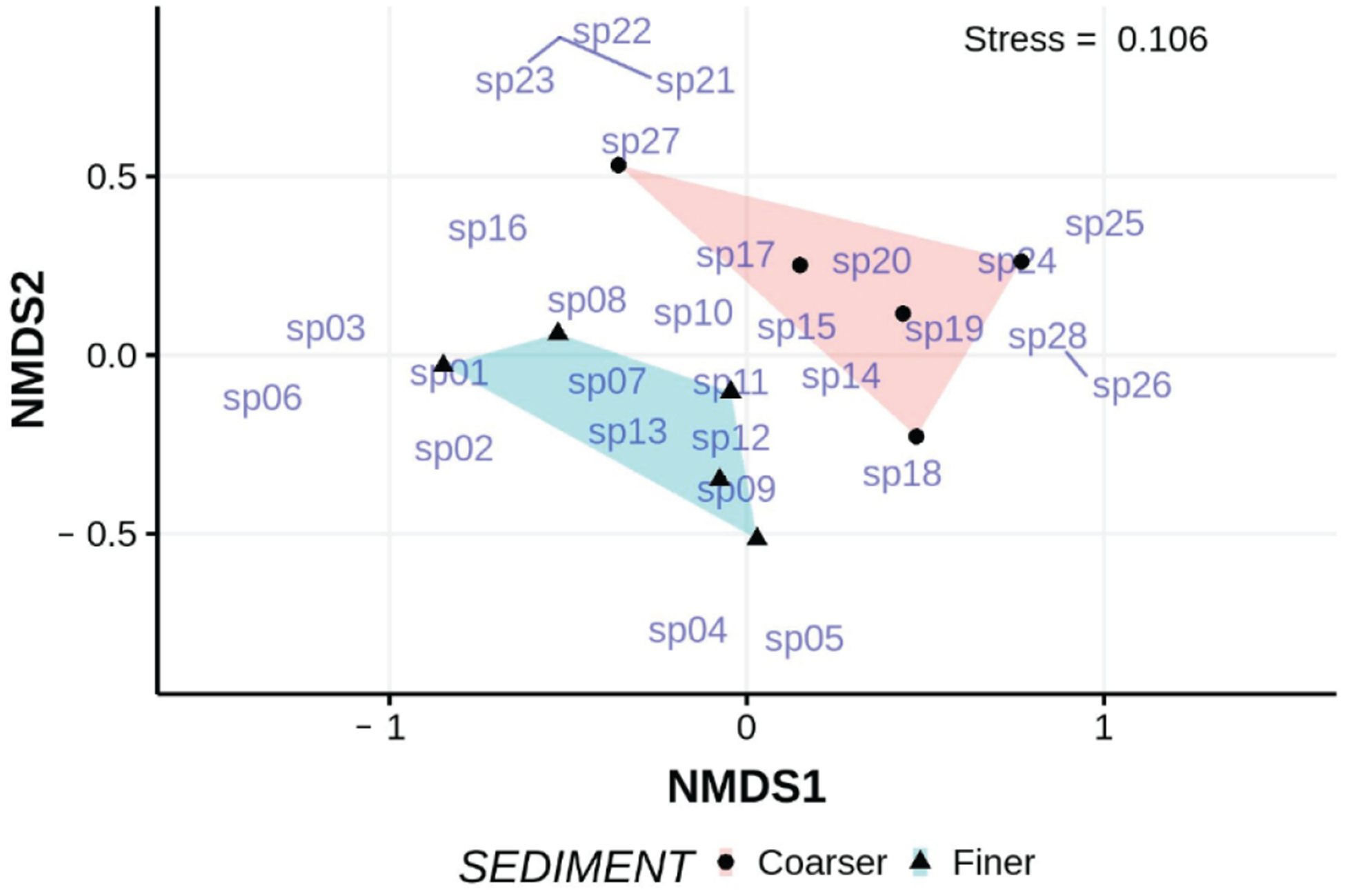
nMDS ordination of the abundances of turbellarian species found in finer and coarser sediments collected from the lower intertidal of EI beach in 2012; non-metric scales serve to ease visual estimation of dissimilarity distances but are not associated with any specific units. Species codes, “sp__”, reference to the sequence of species listed in Table 7. Names of undescribed taxa used here are labels and not being made available for formal taxonomic purposes.

Turbellarian species richness appears considerably higher at the EI site than at ISP (~108–126 spp vs. ~45–47 spp). In one sampling in March 2012, we identified 28 different species of turbellaria from finer and coarser surface sediment samples at EI beach (Table 7). Of these, 6 species were found exclusively in finer sediments and 8 in coarser sediments. Numbers of individuals are too few to conduct meaningful univariate analyses, but PERMANOVA indicated that the turbellarian assemblages differed significantly (*p* < 0.025, homogeneity of multivariate dispersions *p* = 0.94) between finer and coarser sediments (Figure 13). Five species contributed significantly to the overall dissimilarity between finer and coarser assemblages as revealed by SIMPER, with the monocelid “skinny” (13.2%) and *Nematoplana* (8.4%) among the most important.

### 3.4. Meiofaunal Selection for Finer or Coarser Sediments—General Question #3

**Figure 14.**
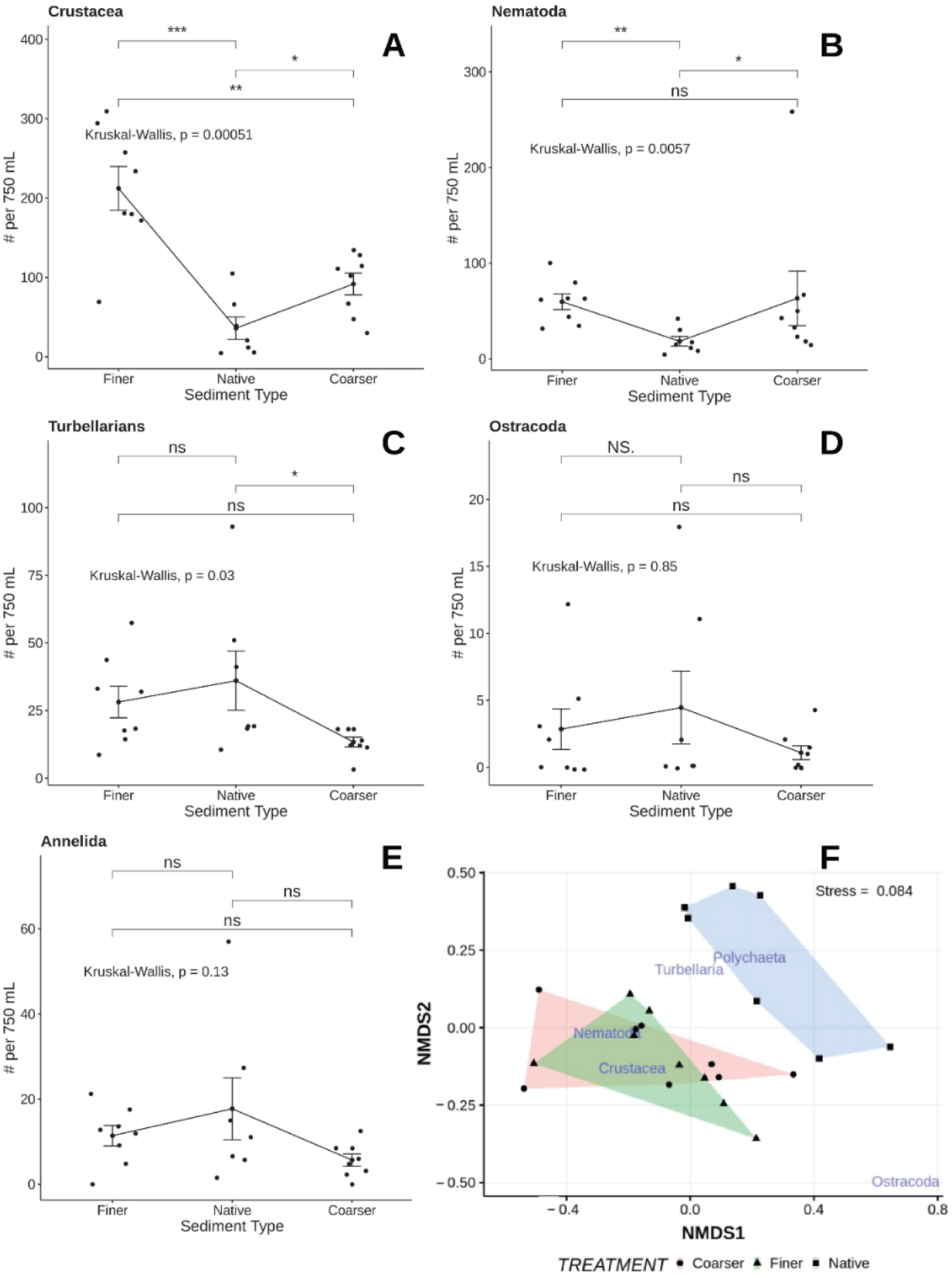
Mean (±1 SE) and observed abundances (dots) for major taxa (**A**–**E**) observed in the different treatments of the sediment selection experiment. Horizontal lines and associated probabilities (above each line) indicate results of separate Kruskal–Wallis comparisons of respective means. nMDS ordination of species assemblages associated with each experimental sediment type shown in (**F**); non-metric scales serve to ease visual estimation of dissimilarity distances but are not associated with any specific units. ^ns^—*p* > 0.05, *—*p* < 0.05, **—*p* < 0.01, ***—*p* < 0.001.

Meiofaunal abundances indicate that different taxa selectively colonized finer and coarser sediments (Figure 14). Copepods were most abundant in fine sediment and intermediate in abundance in coarse sediment compared to native sediment. Nematodes occurred at abundances higher than seen in native sediment in both manufactured sediments, but the latter were not different from each other. Turbellaria were significantly lower in the coarse sediment treatment than either native or fine sediments. Ostracods and annelids showed no differences in abundances among sediment types. Gastrotrichs were not abundant enough to detect any patterns. Multivariate analysis also indicated that the meiofaunal assemblages of both experimental sediment types differed significantly from native sediments, although they did not differ from each other (*p* < 0.001, homogeneity of multivariate dispersions *p* < 0.121); Figure 14). SIMPER indicated that the finer sediments differed because of different copepod abundances (68%) while the coarser sediments differed from native sediments by significant differences in nematode (27.6%), turbellarian (13.7%), and polychaete (3.3%) abundances.

### 3.5. Patterns of Meiofaunal Diversity as Revealed by Metabarcoding—General Question #4

**Figure 15.**
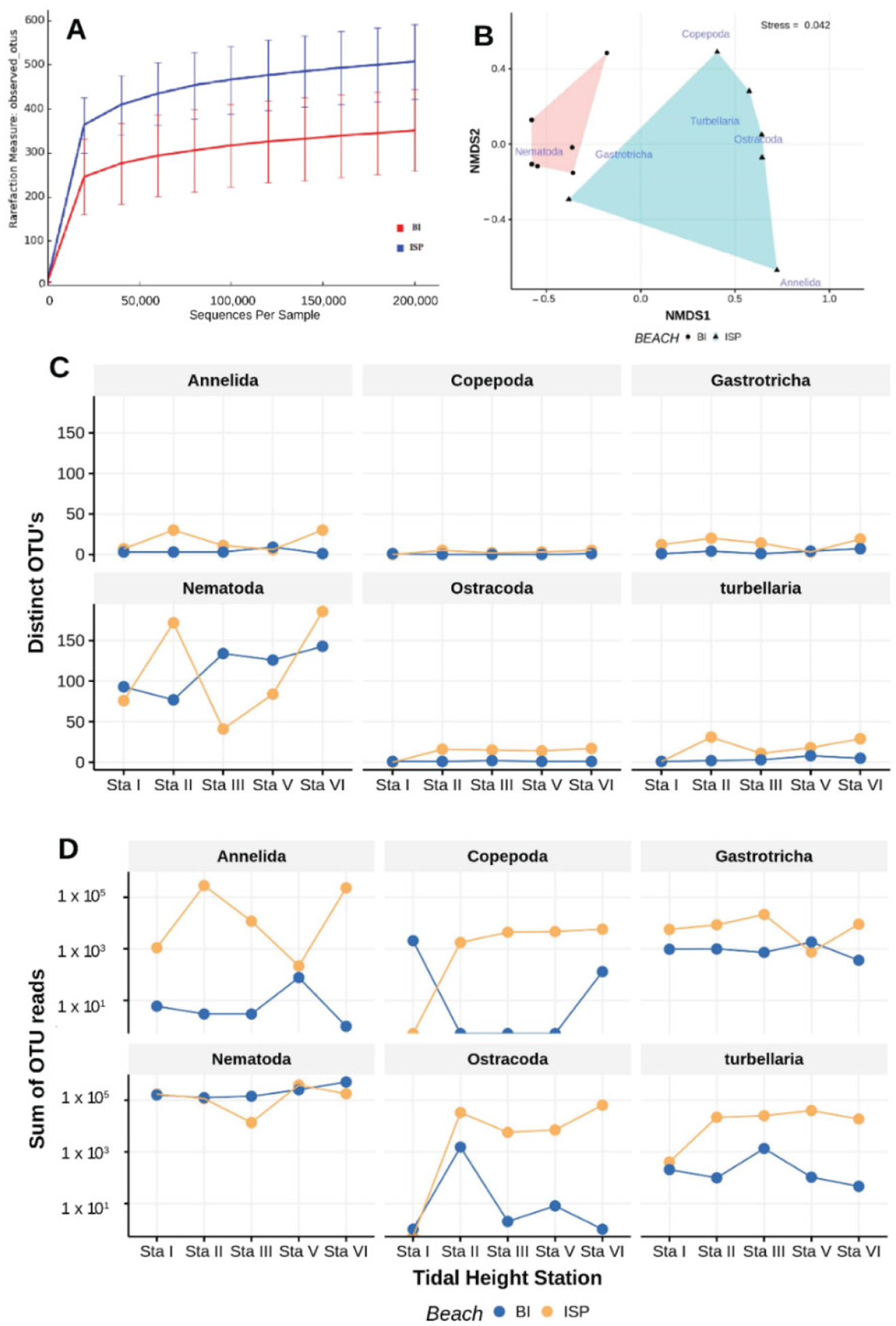
Results of metabarcoding meiofaunal samples collected from unnourished (BI) and previously nourished (ISP) beaches. Rarefaction curves derived from observed OTU’s for each beach (**A**). OTU reads (**C**) and number of OTU’s (**D**) observed at each tidal height (Sta I—MHW, Sta II—intermediate between MHW and MTL, Sta III—MTL, Sta V—MLW, Sta VI—wave swash) for each beach indicated along with probabilities of separate, paired *t*-test comparisons for each beach. nMDS ordination for the OTU assemblages (**B**); non-metric scales serve to ease visual estimation of dissimilarity distances but are not associated with any specific units.

Sediments were finer and better sorted at BI than ISP (respectively, average median phi size = 2.48 and 1.49, Wilcoxon *p* < 0.008; sorting = 2.11 μm and 2.66 μm, Wilcoxon *p* < 0.022). DNA yields per sample were comparatively low, averaging 260 ng total DNA (Picogreen) from 150 cc of sediment. DNA yield from Sta IV (intermediate between MTL and MLWN) for BI proved too low for amplification, so the corresponding sample from ISP was also omitted from analyses. The samples yielded slightly more than 5.1 M quality-filtered reads. Clustering with QIIME open-reference OTU picking produced 31,136 OTUs. In contrast, clustering with Usearch (97%) produced 1540 OTUs, whereas clustering with Unoise (100%) produced 1966 OTUs. Median Read-number for the 12 samples was 327,752. Rarefaction analysis indicated that sequencing depth was adequate for both sites (Figure 15).

Paired *t*-tests revealed a significant difference between ISP and BI OTU richness only for turbellaria, although the *p*-value for gastrotrichs was low (0.086). Except at MHW (Sta. I), more distinct OTU’s were observed at ISP tidal heights. Nematodes demonstrated dramatically different numbers of OTU’s at most tidal heights, but the overall difference between beaches was non-significant. Total OTU reads (abundances) for copepods, ostracods, gastrotrichs, and turbellaria were all higher at ISP than BI (Figure 15). Nematodes showed no overall difference in abundances between the two beaches although a gradual increase in abundance with decreasing tidal height at BI is apparent, but not at ISP. Multivariate analysis indicated a clear and significant (PERMANOVA *p* < 0.04, dispersion *p*< 0.158) distinction in community structure between the two beaches (Figure 15). SIMPER analyses indicated that turbellaria (7.6%), ostracods (6.2%), and gastrotrichs (4%) were most important in distinguishing the communities even though their individual contributions to dissimilarity were low.

Of the 40 turbellarian OUT’s obtained from ISP samples, eight could be identified uniquely to morphospecies by match to their 18S sequences. Four additional congeners to known species could be identified among the remaining OTUs (Table 8).

### 3.6. Responses of Meiofaunal Abundances to Beach Disposal—General Question #2 and #3.

**Figure 16.**
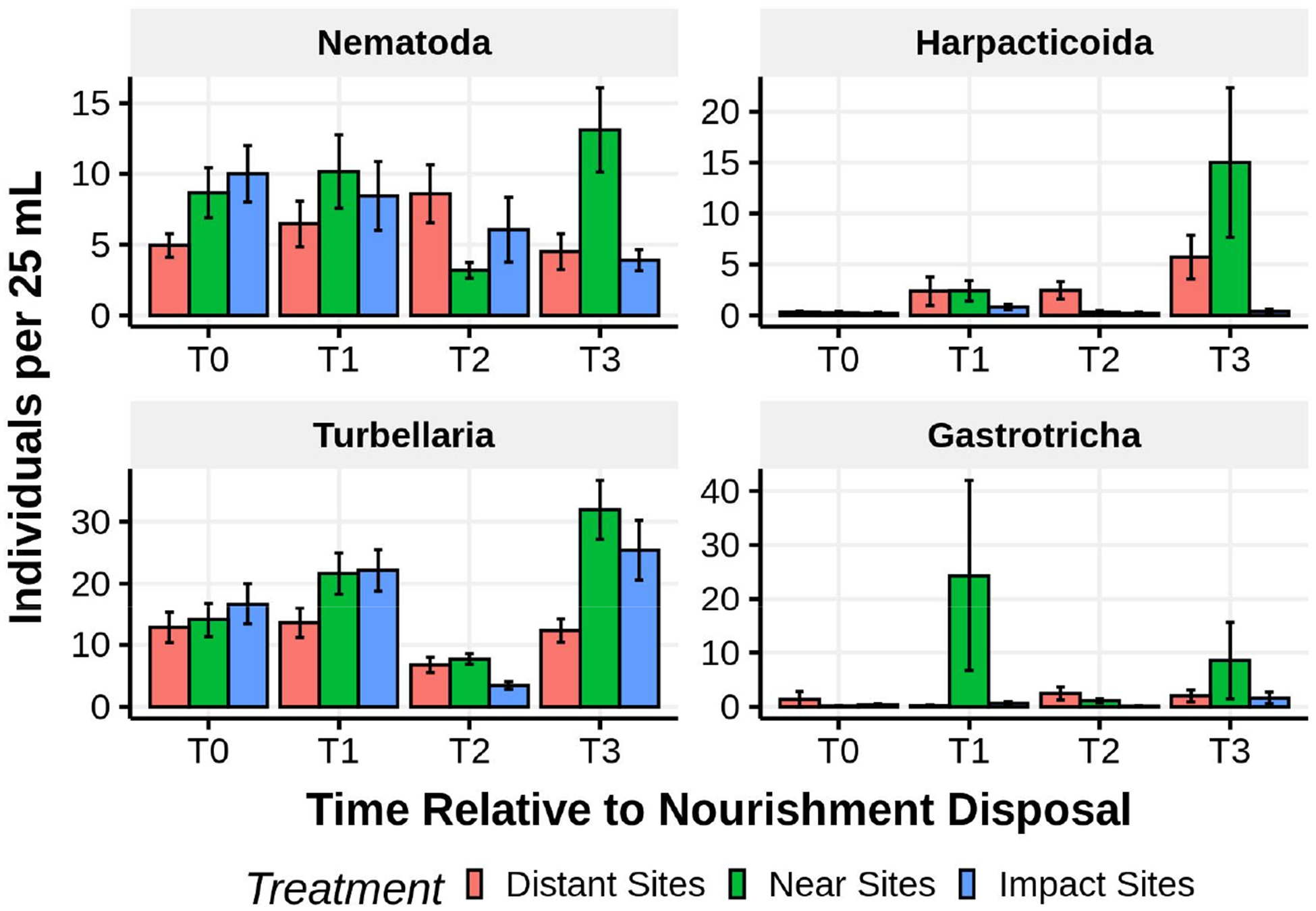
Mean (±1 SE) abundances of major taxa in the lower intertidal of sites directly receiving dredge spoil (impact sites), sites bordering the impact area (near control sites), and sites 3–5 km distant from nourishment activities. Time codes are: T0—before nourishment; T1—during nourishment; T2—the day after nourishment ended; and T3—5 weeks after nourishment ended.

Patterns of meiofaunal abundances at sites receiving different levels of beach nourishment (direct, indirect, and none) varied depending on taxon and time of sampling relative to nourishment (Figure 16). A treatment by time ANOVA (sites nested within treatment and blocks nested within site) revealed significant time by treatment effects for the soft-bodied fauna (turbellaria *p* < 0.0001, gastrotrichs *p* <0.049) but not for either copepods (*p* < 0.521) or nematodes (*p* < 0.299). Within sampling times, gastrotrichs never showed significant differences among beaches. Turbellaria were significantly less abundant, compared to unnourished areas, immediately after nourishment ended in beaches directly receiving nourishment sediments (direct < indirect = none; *p* < 0.015) and showed higher abundances within the indirect beaches 5 weeks after nourishment ended (none = direct < indirect; *p* < 0.009). Nematodes only differed significantly 5 weeks after nourishment ended with both directly and indirectly affected beaches having higher abundances than the unaffected beaches (none < direct = indirect; *p* < 0.029). Harpacticoid abundances differed significantly only among beaches receiving different treatments within a sampling time immediately after nourishment ended (direct = indirect < none; *p* < 0.019) when abundances were depressed on nourished beaches and those adjacent to them.

**Figure 17.**
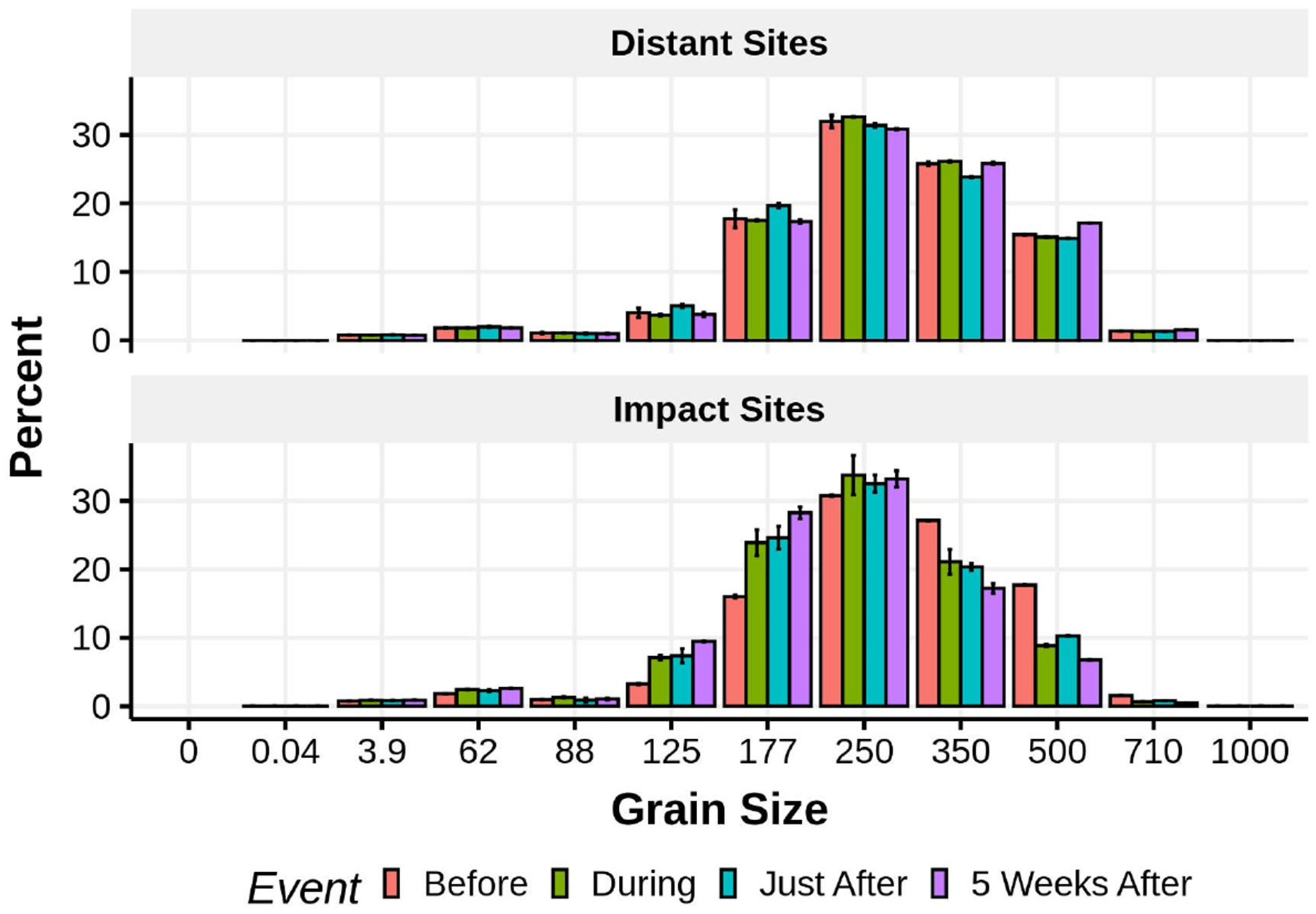
Mean (±1 SE) percent grain size composition of sediment samples taken from the distant and impact sites before, during, just after, and 5 weeks after nourishment.

Comparison of grain-sizes at the distant and impact sites through time (before, during, just after, and five weeks after nourishment) revealed two patterns (Figure 17). At the distant sites grain-size distributions stayed fairly constant over the entire 2 month interval. At the impact sites sediments became finer as the percent composition of 125 and 177 μm grain-size classes increased during nourishment and remained elevated while percent composition of the 350 and 500 μm grain-size classes decreased.

## 4. Discussion

Our studies of meiofaunal abundances and sediment character, within and among exposed barrier island beaches and across time, strongly suggest that beach nourishment, the principal anthropogenic activity affecting beach granulometry in our region, has altered indigenous meiofaunal communities. Differences in sediment granulometry have long been known to alter the porosity, chemistry, food quality, and substrate stability of protected benthic environments in ways that affect both macrofaunal and meiofaunal communities by both direct and indirect means [45,46]. It seems reasonable to expect fauna occurring in exposed, unprotected habitats to be affected by sediment character. Given the occurrence of spatial and temporal variation associated with the distribution of meiofauna, we recognize the limitations associated with comparing contemporary results to those from a very limited number of historical studies. Any differences between the present and the past inferred from a single sample could be a function of natural variation without any state change in community structure. Yet the existence of historical data sets cannot be ignored. In order to draw reasonable conclusions, given the confounding natural variation among studies, we rely on examining multiple comparable studies from the past and present. We do so at both the major taxa-level and, regarding turbellaria, species-level. In addition, we explore whether taxon-specific relationships between exposed beach intertidal meiofauna and grain size characteristics exist. In sum, these approaches give the best opportunity to decide whether meiofaunal communities have changed since the pre-nourishment era due to, at least in part, changes in sediment structure over that time.

### 4.1. Sedimentary Structure of the Beaches

We found beach sands at several locations on Bogue Banks appeared coarser and more heterogeneous than they were in the era prior to nourishment. Previous studies have documented altered grain sizes and sorting within Bogue Banks beaches in the months to years following nourishment. In a minority of cases the changes reduced median grain size when spoil sediments were a poor match to native sediments [10], but generally beach sediments were coarsened by nourishment [12,14,15,23]. What surprised us was the evidence of elevated very coarse sediment and shell-hash fractions with increasing vertical depth into the beach. This pattern is strikingly different from the findings that historical studies found at depth in these beaches [27–30]. As with other exposed beaches, the top 20–30 cm of surface sediments on Bogue Banks beaches are dynamic and can be rapidly eroded or replaced by natural sediment transport processes [21,47]. Below this surface stratum shell hash, heavy minerals, and larger sand grains can accumulate. However, grain size is one of the factors affecting sediment activation depth [48,49] with coarser sediments thinning the active zone of surface transport and mixing. Because Bogue Banks beaches are not in equilibrium currently, due to continued local relative sea level rise [50], finer sediments will continue to winnow away as the beaches erode and coarser heavier materials will be selectively retained at depth. However, future nourishments are likely locally [51], which will reintroduce finer sediments to the beach that would maintain heterogeneous beach sediment structures.

The consequences of decade-long shifts in sediment composition to coarser materials are substantial. Changes in sediment composition can alter beach morphology (e.g., slope steepness, width, profiles), interstitial porosity and chemistry, sediment penetrability, and sediment stability, all of which affect benthic meiofauna and macrofauna directly and indirectly [18,52]. In addition, we argue that long-term accumulation of coarse materials in the sediment has provided a structurally more heterogeneous environment for meiofauna in Bogue Banks beaches. Personal observations by the two lead authors contrasting surface sediments of these beaches between the late 1970s and the present suggested patches of coarse material embedded among finer sands were more numerous and widespread. The granulometric results of our contemporary studies that selectively sampled visually finer and coarser surface patches of sediment confirmed our ability to detect such patches. Furthermore, the results of both the 2013 and 2014 studies revealed the existence of multiple strata of finer and coarser sediments with depth, which differs from what Lindgren [28] documented. The physical structure of these beaches exhibit increased spatial heterogeneity of coarse sediments.

### 4.2. Relationship of Exposed Beach Meiofauna to Finer and Coarser Sediments

We observed considerable variation among the results of our individual studies in the relationship between abundances of major taxa and whether sediments were finer or coarser. This is not surprising because granulometry has long been recognized as one among several environmental factors influencing meiofaunal diversity and abundances [18,53]. However, some patterns emerge across the array of our results.

Of the four numerically dominant taxa, gastrotrichs appear as the exposed beach group most likely to be altered by sediment coarsening. With very few exceptions, gastrotrichs were least abundant where sediments were coarsest or sorting was poor. Indeed, in our most recent studies in 2013 (sediment with depth at ISP) and 2016 (cage study at ISP) gastrotrichs occurred in numbers so low or were spatially so restricted that they would no longer be considered a dominant taxon. Metabarcoding indicated that gastrotrichs were significantly higher in abundances and species richness at ISP, which has been nourished, versus unnourished BI, but a sampling conducted in 2006 by one of the authors (SRF) found that mean (±1 SE) gastrotrich abundance between those two sites was 8× lower at BI than ISP (6.0 ± 1.3 versus 49.9 ± 16.8 gastrotrichs per 25 mL), so it is difficult to conclude that a change in relative abundances has occurred. Comparison between the 1969 and 2013 ISP studies found that gastrotrich abundances did not change at most tidal heights, but comparison of the 1976 and 2012 studies reveals a dramatic decrease in gastrotrich abundance. Although the pattern of gastrotrich abundances with sediment character is not consistent, we found coarser sediments generally harbor lower gastrotrich abundances.

The other dominant soft-bodied group, turbellaria, and both hard-bodied taxa, copepods and nematodes, demonstrated variable patterns associated with sediment character, suggesting that individual species within these taxa may be affected differently by differences in median grain size and sorting. Some individual species may prefer coarser material while other are more abundant in finer, better sorted sediments. If true, this would explain the higher abundances and richness for these taxa indicated by metabarcoding results in the comparison of the granulometrically more diverse ISP to the more uniform BI. However, our 2013 study of sediment character by depth results indicate that all of these taxa were less abundant with depth if sediments coarsened and were more poorly sorted. Rieger’s unpublished results and several published historical studies at ISP [26–30] revealed that copepods, gastrotrichs, *Derocheilocheris*, and turbellarian species maintain specific distributions dependent on salinity, tidal stage, and depth from the surface, which has been observed elsewhere [54]. In all of the ISP studies the sediment character during the late 1960s and early 1970s was more uniform with depth than it is currently at ISP. Current sediment structure at ISP may be altering how meiofauna exploit their habitat by introducing new patches of coarse material combined with losses in communication between patches of finer material. The results of the cage study do indicate that copepods, nematodes, and turbellaria do respond differently to sediment composition, over short time scales.

Specific responses of meiofauna to beach nourishment was highly variable across taxa, treatment, and time. The effects that were seen were limited to times during and immediately after nourishment. Samples taken during nourishment may underestimate the effect of beach disposal to native meiofauna, because meiofauna from the subtidal Bogue Sound source of the disposal sediments almost certainly were transported to the beach [55,56]. Examination of the samples at the major taxon level would not be able to distinguish between exposed beach species and imported subtidal, sound species. Alterations to sediment structure were modest during this disposal and, during and in the aftermath, produced a fining of the sediments. Given that the volume of sediment placed on the beach was relatively small compared to the beach nourishments that occurred on this island in the 2000s and it did not coarsen the sediments over the short-term, it may not serve as a representative guide to meiofaunal responses during the nourishment of the entire island in that decade. Changes of meiofaunal abundances in the impact area immediately after nourishment ended were likely a function of burial and physical disturbance associated with sediment emplacement and bulldozing [57], as well as the introduction of estuarine species. In addition, abundances at the impact site and nearby locations may have been enhanced several weeks after nourishment activities ended as a result of the pulse of organic material added to the beach from the sound sediment source.

### 4.3. Community Effects of Beach Coarsening

Our DNA yields were far lower than predicted based on the method we used; published work using this method resulted in 10-fold higher yields at minimum (Brannock, pers. comm., Appendix B). Despite the lower than expected DNA yields, the tendency for OTU numbers and types between ISP (nourished) and BI (unnourished) beaches to favor the nourished beach (except for nematodes) seemed clear. These patterns cohere with the general results we observed in our studies reliant on microscope-based methodology. With the exception of gastrotrichs, abundances of major taxa and turbellarian richness are enhanced in beaches that have experienced nourishment when compared to historical results or to locations receiving less nourishment. When we estimate the differences in meiofaunal community structure with sediment character, across time and space, at both the major taxa and putative species levels, ordination revealed distinct, significant groupings coupled to finer or coarser sediments. Such an effect on beach meiofaunal community structure has also been documented recently in a study comparing a range of tourist-impacted beaches where sediment granulometry was a significant factor affecting species richness [58]. Our groupings may also be a function of changes to grain size sorting, which often varied among our studies, as this aspect has been shown to influence nematode abundances [20]. The taxa defining the distinct groupings are not consistent, but in the majority of cases copepods significantly contributed the most to group dissimilarity, followed closely in importance by gastrotrichs: the former tended to be higher in abundance in coarser sediments, the latter lower in abundance. We have demonstrated as well that changes in sediment–faunal relationships extend to depth into the beach (Figures 8 and 12).

Armonies [59,60] repeated sampling of historical studies to investigate changes in turbellarian assemblage structure over time for a stable intertidal beach and a dynamic tidal inlet. At the former site he found little evidence for change. At the latter, he found substantial site-specific changes in assemblage structure. However, he found that specific locations in the tidal inlet no longer demonstrated the same physical parameters as they had historically and when he found contemporary sites comparable to historical sites the turbellarian assemblages were similar. Using results from both historical-contemporary comparisons and comparisons from beaches with alternative nourishment histories, we argue that meiofaunal community structure has changed because the exposed, intertidal beach habitat has been physically altered directly by anthropogenic activities and relative sea level rise. Similar to the results of Baldrighi et al. [61], who investigated sandy beach meiofaunal responses to macroalgal blooms, our results are not always consistent; high spatial variability of meiofauna, the presence of multiple microhabitats, and relatively low intensity replication constrain the results of many meiofaunal studies.

Considering the numerous studies documenting negative effects on beach macrofauna, fish, and birds (cited in the introduction), our general results were unexpected. We hypothesize that observed increases in beach meiofaunal abundances and richness result from increased habitat heterogeneity provided by relative increases in the amount and patchiness of coarser sediments over historical conditions. Meiofauna disperse, actively or passively, over greater distances and with higher frequency than had been assumed previously [62]. The coarser “islands” of sediments that persist in nourished beaches could recruit and maintain species more typically associated with coarser habitats typical in inlets and shallow subtidal locations. To test this hypothesis, we will need to complete species-level surveys of beach meiofauna, based on both genetic and morphological information, and determine species-specific associations with *in situ* alternative sediment types.

In addition, the patterns we observed in sediments and fauna need to be examined with respect to other environmental changes affecting beach meiofaunal communities including increased intensity of tourist visitation [58] and the array of environmental factors identified by Zeppilli et al. [63] that can affect shallow water systems such as increasing water temperatures, changes in primary production, ocean acidification, and introduction of alien species. We did not measure or record any of these alternative factors in our study and cannot speculate on relative impacts among all the factors changing in our beaches. In addition, too little is known of both the structure and dynamics of outer- beach meiofaunal communities to allow us to parse whether granulometric changes alter meiofauna directly or through mediation of trophic relationships. We suggest that the hypothesized existence of feeding guilds in both gastrotrichs and flatworms [64], be tested using diagnostic PCR [36,65,66] to elucidate the likely pathways that changes in individual species may have on beach assemblages.

## 5. Conclusions

We do not argue whether changes in meiofaunal assemblages in exposed beaches are good or bad regarding beach ecosystem function. However, we do assert, due to a persistent alteration of beach sediment composition, that there is evidence that faunal changes have occurred which could alter beach function. The suggestion that meiofauna play functional roles in beaches important to provision of beach ecosystem services was made decades ago [67]. Although the roles of beach meiofauna in recycling nutrients and influencing beach bacterial populations have not been quantified extensively and examined under a range of conditions, several studies have demonstrated that meiofauna are a functionally important beach community component [19] and provide useful information for policy makers [68]. Unfortunately, changes to the physical environment of the beach, arising from anthropogenic activities [58,69–72] and responses to shoreline erosion, may be altering beach meiofaunal communities in ways that affect beach processes, before we fully understand those processes.

## Supplementary Material

1

## Figures and Tables

**Table 1.** Summary of the individual characteristics of the multiple meiofauna sampling projects conducted on Bogue Banks beaches that are reported in this study. Beaches: EI—Emerald Isle; ERBA—Eastern Regional Beach Access; FM—Fort Macon; SR—Spinnaker’s Reach; ISP—Iron Steamer Pier; PKS—Pine Knoll Shores; BI—Bear Island (not located on Bogue Banks). Tidal heights: MHW—mean high water; MWHN—mean high water neap; MTL—mean tide level; MLWN—mean low water neap; MLW—mean low water; STL—shallow subtidal; “Sediment-Selected” indicates whether equal numbers of replicate samples were collected after visually selecting for patently finer or coarser sediments. Numbers in the “Notes” column indicate which questions, defined in the introduction, were addressed by the respective study.

Year	Beach	Tidal Heights	Sediment Depths (cm)	Sampled	Sediment-Selected?	Notes	Source
1969–1970	ISP	supra- to sub-littoral	surface to water table	sediment, fauna	no	1	Lindgren, PhD thesis
1969–1970	EI, ISP	MHW–MLW	depended on tidal height	sediment, fauna	no	1	Rieger, Unpublished
1976	EI	MTL, MLWN	depended on tidal height	fauna	no	1 — resin slides	Rieger, Unpublished
2006	SR, ERBA, ISP, FM	MLW	0–10	sediment, fauna	yes	2	Fegley, this paper
2008	PKS, AB	MLW	0–10	sediment, fauna	no	2	Fegley, this paper
2012	EI	MTL	0–5	sediment, fauna	yes	1, 3, 4—resin slides^1^	Smith III, this paper
2012	EI	MTL	0–5	sediment, fauna	yes	3—microscope	Smith III, this paper
2012	EI	MHW–MLW	0–2, 2–4, 4–10	sediment	no	1 — for 1970 comparison	Smith III, this paper
2013	ISP	MHW–MLW	depended on tidal height	sediment	yes	1, 3	Smith III, this paper
2013	ISP	MHW–MLW	surface to water table	sediment, fauna	yes	1, 3 — for 1969–70 comparison	Fegley, this paper
2014	ISP	MTL–MLWN	depended on tidal height	sediment, fauna	yes	3	Fegley, this paper
2017	ISP, BI	MHW–sublittoral	depended on tidal height	sediment, fauna	no	2, 4—metabar-coding	Smith III, this paper

1 100 mL samples used.

**Table 2.** Results of separate, 2-way (year versus tidal heights) Scheirer–Ray–Hare tests comparing faunal or granulometric variables collected in a 1969 study and a repeat of that study in 2013. Probabilities < 0.05 are in bold.

Variable	Year	Tidal Ht.	Year × Tidal Ht.
Copepoda	**0.029**	0.139	**0.009**
Nematoda	0.067	0.192	0.436
turbellaria	**0.014**	**0.001**	0.371
Gastrotricha	0.232	**0.001**	0.486
median grain size	**0.004**	0.628	0.829
grain sorting	0.249	0.213	0.076

**Table 3.** Comparison of turbellarian species found within the last decade at ISP to those in collections made in the same location or nearby beaches in the 1970s. “BB” = found by Rieger at other sites on Bogue Banks; “nf” = not found; “OTU” = not observed alive, but present in OTUs from ISP (see section 3.5); “spp.” = at least two congeners appear in metabarcoding data. Names of undescribed taxa used here are labels and not being made available for formal taxonomic purposes.

Clade	Rieger 1970 Survey	Contemporary Species List
Catenulida	BB	*Retronectes atypica*
Acoelomorpha	*Anaperus gelb*	*Anaperus singularis*
	nf	*Haploginaria schillingi*
	nf	*Praeconvoluta* cf. *tigrina*
	BB	*Paratomella rubra*
Macrostomorpha	*Myozona gelb*	*Myozona* ISP spp.
	BB	*Paromalostomum* sp.
	nf	*Psammomacrostomum* spp. (OTU)
	BB	*Haplopharynx* cf. *rostratus*
Prolecithophora	*Plagiostomum* sand	*Plagiostomum* “*corculum*”
Proseriata	Otoplanid 1	*Parotoplana* “stately Oto”
	Otoplanid II	*Kataplana celeretrix*
	BB	*Prosogynopora riseri*
	*Paramonotus* sp.	Monocelididae n.g., n.sp
	*Archimonocelis* ISP	Monocelidid 4-testes
	*Monocelis bitestis*	Monocelidid 2-testes
	*Nematoplana*	*Nematoplana* spp.
	*Polystylifora* sp.	*Polystyliphora* cf. *karlingi* spp.
	nf	*Prosogynopora riseri*
	nf	*Cirrifera* cf. *xanthoderma*
Rhabdocoela: Kalyptorhynchia	Cicerina “orthocirri”	*Cicerina debrae*
	*Cheliplana* sp.	*Cheliplana* “blind October”
	*Cheliplanilla* ISP	*Cheliplanilla* “schwanzi”
	*Carcharodorhynchus* ISP	*Carcharodorhynchus* “ungleich ISP”
	nf	*Carcharodorhynchus* “small”
	*Thylacorhynchus* “*schwanzi*”	*Thylacorhynchus* “schwanzi”
	*Schizorhynchoides* “*atoptus*”	*Schizorhynchoides “lupus”*
	*Proschizorhynchus* “*faeroennsis*”	*Carolinorhynchus follybeachensis*
	Kalypto macropharynx	*Lehardyia alleithoros*
	nf	*Lehardyia* sp. 2
	Kalypto blind ISP	nf
	nf	*Karkinorhynchus* “*carolinensis”*
	Proschizo juvenile	*Proschizorhynchella “shaunae”*
	*Uncinorhynchus* sp.	*Drepanorhynchides* cf. *hastatus*
	*Neognathorhynchus* sp.	*Gnathorhynchus “caudafiliformis”*
	Eukalypto spitz	*Placorhynchus* cf. *doei?*
	Eukalypto spirale	nf
	Eukalypto riese	Eukalypto riese
	nf	*Cystiplana* cf. *rubra*
	BB	Eukalypto schrag
Rhabdocoela: Dalytyphloplanida	Promesosto juvenile	*Promesostoma* ISP
	Russekopf ISP	*Coronhelmis* sp.
	BB	Dalytyphloplanida n.gen., n.sp.
	BB	Dumpy typhloplanid

**Table 4.** Results of separate, 2-way Scheirer–Ray–Hare tests comparing faunal or granulometric variables collected from four Bogue Bank beaches spanning the length of the island with each beach having unique nourishment histories. Probabilities < 0.05 are in bold.

Variable	Beach	Sediment Type	Beach × Sediment Type
Copepoda	**0.008**	0.347	0.888
nauplii	**0.010**	0.807	0.635
Nematoda	0.258	0.530	0.493
turbellaria	**0.009**	0.830	**0.011**
Gastrotricha	**0.034**	0.890	**0.017**
median grain size	**0.002**	**0.001**	0.919
grain sorting	**0.002**	**<0.001**	0.665
percent gravel	**0.006**	**<0.001**	0.590

**Table 5.** Mean (±1 SE) density (# individuals per 25 mL) of each major taxon found in visually distinct finer and coarser surface sediments from the lower intertidal of EI. Probabilities derive from separate, Mann–Whitney comparisons testing the null hypothesis of no difference in faunal abundances between finer and coarser sediments. *n* = 5 for each sediment type. Significant results (*p* < 0.05) are in bold.

Taxon	Finer Sediment	Coarser Sediment	Probability
Archiannelida	5.6 (1.4)	0.6 (0.3)	**0.011**
Crustacea	20.0 (3.9)	98.8 (41.5)	0.144
Nematoda	47.2 (6.9)	27.8 (4.5)	0.075
Turbellaria	43.8 (7.5)	32.4 (3.2)	0.347
Gastrotricha	31.2 (10.4)	1.6 (1.1)	**0.012**

**Table 6.** Parameters of linear regressions of ln-transformed abundances of major taxa onto depth with sediment type as a cofactor. When the interaction of the overall model is significant the parameters of the separate regressions (finer or coarser) are presented.

Taxon	Sediment × Depth	Variable	Intercept	Slope
Copepoda	0.038 *	Finer	2.26 ***	0.003 ^ns^
		Coarser	2.59 ***	−0.049 ***
Nematoda	0.0004 ***	Finer	2.32 ***	0.004 ns
		Coarser	3.09 ***	−0.045 ***
turbellaria	0.0024 **	Finer	0.76 ***	0.046 ***
		Coarser	2.45 ***	−0.009^ns^
Gastrotricha	0.66^ns^	Depth	0.25 *	0.017 *
		Sediment type	0.25 *	−0.231^ns^
		Sediment type × Depth	0.25 *	0.006 ^ns^

ns— *p* > 0.05,

*— *p* < 0.05,

**— *p* < 0.01,

***— *p* < 0.001.

**Table 7.** Turbellarian species (undescribed species listed with helping names) found in finer and coarser sediments in the lower intertidal of EI in March 2012. Numbers represent the mean (±1 SE) number of individuals per 25 mL.

“Species”	Finer Sediments	Coarser Sediments
Eukalypto unlD’d	2.8 (2.1)	0
Oto2/Oto3 undetermined	2.6 (2.1)	0
Gnatho Schwanzi	0.6 (0.4)	0
Polycystididae	0.4 (0.2)	0
Macrostomorpha (not *Myozona*, *Microstomum*, or *Paromalostomum*)	0.2 (0.2)	0
Eyed Eukalypt small	0.2 (0.2)	0
Otoplanid n. sp. “hannahfloydae”	8.4 (2.1)	5.0 (1.8)
Immature Oto	6.6 (2.1)	4.4 (1.7)
“Monocelid” (skinny)	6.2 (1.3)	1.0 (0.6)
Eukalypto Zange	4.8 (1.7)	2.2 (1.3)
Stubby	4.0 (0.9)	4.0 (0.3)
*Polystyliphora* spp.	1.8 (0.4)	1.4 (0.4)
Unknown proseriate	1.2 (0.5)	0.2 (0.2)
Oto straight bundle	1.2 (0.6)	2.6 (0.7)
*Thylacorhynchus* “schwanzi”	1.0 (0.3)	2.6 (0.8)
Eyed Typhloplanoid (immature)	0.4 (0.2)	0.2 (0.2)
*Drepanorhynchus hastatus*	0.4 (0.4)	0.4 (0.2)
Schizo 2-belt	0.4 (0.2)	1.2 (0.7)
EukalyptoSpitz	0.2 (0.2)	0.4 (0.2)
*Nematoplana* spp.	0.2 (0.2)	3.4 (0.5)
TTK	0	0.2 (0.2)
*Diopisthoporus gymnopharyngeus*	0	0.2 (0.2)
*Carolinorhynchus follybeachensis*	0	0.2 (0.2)
Odd little guy (Neodalyellioida)	0	0.2 (0.2)
UnlD’d typhloplanoid	0	0.2 (0.2)
*Plagiostomum* “*corculum*”	0	0.4 (0.2)
Large flat acoel	0	0.6 (0.4)
*Cicerina debrae*	0	0.8 (0.4)

**Table 8.** Selected turbellarian zero-radius OTUs, from ISP or BI, that matched our in-house database of local 18S sequences, showing top blast hit, match to morphospecies for which we have 18S sequences, and four cases (shaded) of congeneric pairs occurring on these beaches. OTUs clustered with -unoise3, as implemented in Usearch 64 v.10. GenBank Blast assignment as of 4.17.20.

OTU #	Top Blast Hit (GenBank)	ISP/EI Local 18S Database Hit	OTU to 18S
387	*Catenula lemnae* isolate K04_69 536/564 2 gaps	*Retronectes atypica* Doe & Rieger	369/369 bp
36	*Coelogynopora tenuis* isolate CTHE1 555/561 1 gap	*Cirrifera* cf. *xanthoderma*	561/561 bp
152	*Monoceolopsis otoplanoides* isolate MOHE1 515/563 4 gaps	Monocelididae n.g. “Stubby”	562/562 bp
158	*Myozona lutheri* voucher MTP LS 692 550/563	*Myozona* n.sp “Myozona ISP”	562/563 bp
719	*Myozona lutheri* voucher MTP LS 692 550/563	*Myozona* n.sp. 2	548/563 bp against above
35	*Nematoplana coelogynoporoides* isolate NCRO2 545/561 3 gaps	*Nematoplana* n.sp. “Nematoplana ISP”	561/561 bp
45	*Nematoplana coelogynoporoides* isolate NCRO2 545/561 3 gaps	*Nematoplana* n.sp. 2	557/561 bp against above
57	*Parotoplanella progermaria* isolate PLBA1 539/561 2 gaps	Parotoplaninae n.sp “Stately OTO”	549/549 bp
58	*Polystyliphora karlingi* McDaniel 551/560	*Polystyliphora* cf *karlingi* EI	560/560 bp
126	*Polystyliphora karlingi* McDaniel 550/560	*Polystyliphora* n.sp. 2	550/560 bp against above
*	*Psammomacrostomum* sp. 1 TJ-2015 558/563	*Psammomacrostomum* sp EI “MCMPH”	
253	*Psammomacrostomum* sp. 1 TJ-2015 558/563	*Psammomacrostomum* n.sp. ISP	548/563 bp against above
795	*Schizorhynchidae* sp. 3 JPS-2015 550/564 4 gaps	*Karkinorhynchus* n.sp. “ESTPG”	561/562 bp

* Specimen collected from Bogue Inlet, photo-vouchered, and sequenced.

## Appendix A

**Table A1.** Taxonomic and non-taxonomic group names used in the text.

Name	Phylum, Subtaxon or Subtaxa
archiannelids	Annelida
Copepoda	Arthropoda, Crustacea
*Diurodrilus*	Annelida
Gastrotricha	Gastrotricha
Harpacticoida	Arthropoda, Crustacea, Copepoda
*Micropthalmus*	Annelida
Mystacocarida	Arthropoda
nauplii	larval Copepoda
Nematoda	Nematoda
Ostracoda	Arthropoda, Crustacea
Polychaeta	Annelida
Tardigrada	Tardigrada
turbellaria (in part)	Xenacoelomorpha, Acoelomorpha
turbellaria (in part)	Platyhelminthes

## Appendix B

### Considerations for Interpreting Metabarcoding Results

Clustering in QIIME drastically inflates the number of OTUs. Among the 31,136 OTUs generated by clustering in QIIME, 1088 were assigned to turbellaria. However, historical records from the Rieger lab and over a decade of contemporary morphological observation suggest that total flatworm species from our sites number 10% to 20% of that total. Clearly, a significant (but presently unknown) proportion of these OTUs is spurious. As others have found, clustering in Usearch provides a better match between OTUs and biological species than does clustering in QIIME [73].

V4/V5 metabarcoding allows identification of some taxa to species-level. The longer amplicon provided by V4/V5 (520 bp to over 600 bp) potentially allows one to relate an OTU to a morphospecies. However, as can be seen from Table 8, the current GenBank database is inadequate for such precise identification, with incorrect generic placement (*Retronectes atypica*) or genetically different congeners assigned to the same Genbank sequence (genera *Myozona*, *Nematoplana*, *Polystyliphora*, and *Psammomacrostomum*). Hence, it is essential that comprehensive local databases of 18S sequences be established. Without these, it is impossible to relate metabarcoding data to morphospecies, and thereby, to ecological information such as diet [36,74].

Improved databases are needed. Although metabarcoding has the potential to uncover new taxa that have not been detected with morphological observation [75–77], actually knowing what to look for depends both on a close match between OTUs and biological species (else one wastes a lot of time looking through duplicate OTUs) and on an adequate database for the amplicon used. Our data indicate that V4/V5 is capable of distinguishing between congeners, and of those shown in Table S1, only one (*Myozona* n.sp. 2) was previously recognized by morphological observation [78].
