# Supplementary material for: Nourished, Exposed Beaches Exhibit Altered Sediment Structure and Meiofaunal Communities
Source: Diversity (Basel). Author manuscript; Available in PMC 2021 Jun 23. (PMC8221582; doi:10.3390/d12060245)
Supplement: 1 [file NIHMS1604467-supplement-1.pdf]

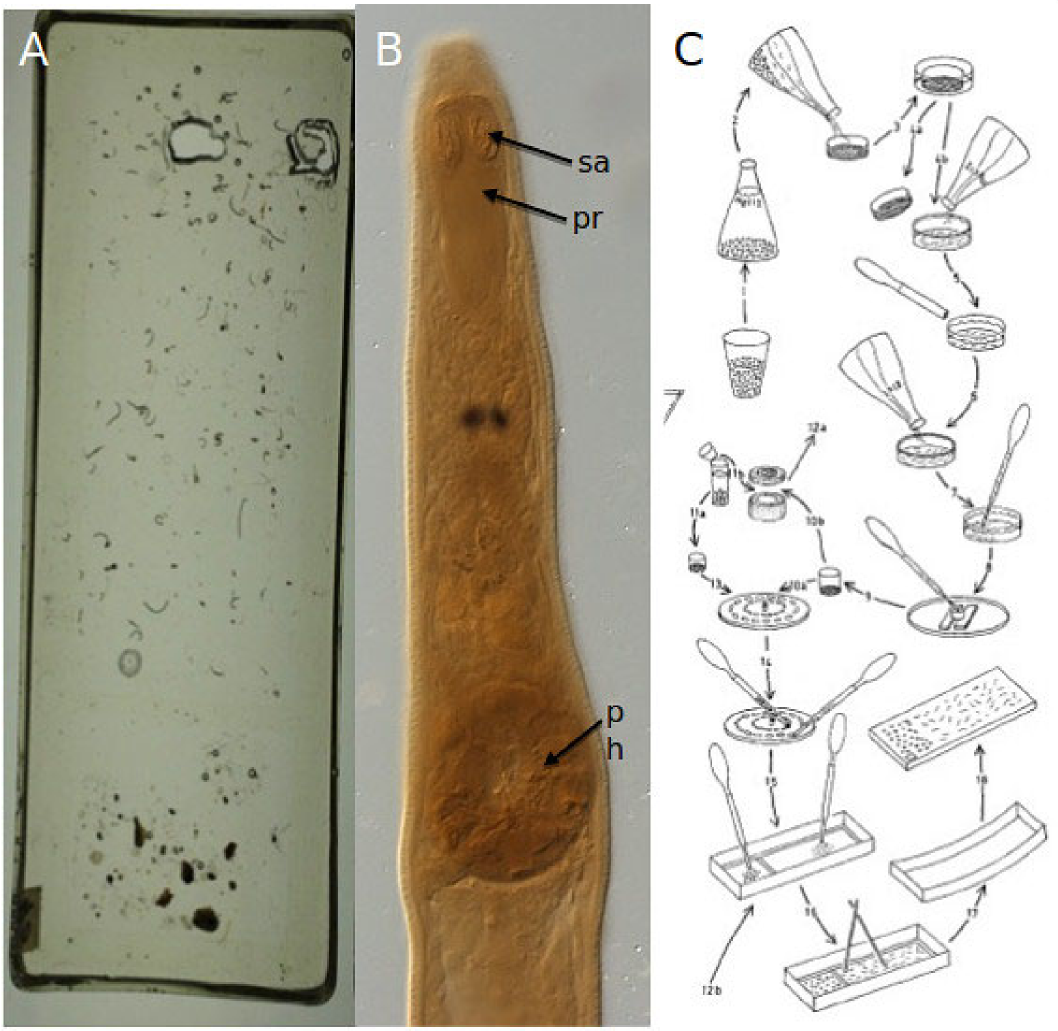

Figure S1. A. Resin slide of whole-sample meiofauna extraction fixed for TEM and embedded in the form of a microscope slide. B. Anterior end of *Cicerina debrae* (Platyhelminthes, Kalyptorhynchia) from such a slide, photographed by DIC microscopy; note proboscis (pr) with secretory ampules (sa) and pharynx (ph). C. Flow diagram of the preparation of resin slides (from 32).

Table S1. Historical/modern comparison of turbellarian species at EI, organized by sub-taxon. Historical data collected by Reinhard Rieger in August 1970 (5500 specimens, ~69 spp). Modern data from collections made from June 2009 – January 2019. Names of undescribed taxa used here are labels and not being made available for formal taxonomic purposes. Abundances are from 1970.

Rieger Helping Name	Abundance	Contemporary name
**Acoeolomorpha (4 spp.)**		
AcoelGelb	37	*Kuma flava*
AcoelBraun	5	*Anaperus singularis* ?
Nemertoderma	7	*Sterreria cf.psammicola*
Paratomella	4	*Paratomella rubra*
AcoelUndet.	23	
**Catenulida (1 sp.)**		
Retronectes outerbanks	10	*Retronectes atypica*
**Macrostomorpha (7 spp.)**		
Microstomum	1	*Microstomum* "gracile"
Paramyozonaria		*Paramyozonaria rosacea* ?
Macrostomum	1	
Myozona	3	*Myozona* sp.

Psammomacrostomum	6	*Psammomacrostomum* n.sp.
Paromalostomum	286	*Paramalostomum* n.sp. "riegeri"
Haplopharynx	9	*Haplopharynx* n.sp.
**Prolecithophora (3 spp)**		
Archimonotresis	3	*Archimonotresis* n.sp.
Plagiosand	215	*Plagiostomum* "corculum"
Plagiotief	3	
**Proseriata (20 spp.)**		
Pros. reise	531	*Cirrifera* cf. *xanthoderma*
Pros. mittelpharynx	8	
Pros atypisch	420	
Pros bitestes	190	Monocelid 2-testes ?
Pros unmoglich	1	*Prosogynopora riseri*
Pros neu	2	
Nematopl gr. Aug	150	*Nematoplana* Augi
Nematopl (unreadable) Aug	40	*Nematoplana* sp.
Togarma	8	
Polystyliphora	407	*Polystyliphora* cf. *karlingi*
Otostumpf	590	*Kataplana celeretrix*
Otospitz	600	*Phyllosyrtis* n.sp. "hannafloydae"?
Otokurzspitz	50	
Otolong	81	
Otogross II	8	Otogross
Otoriese	11	Otoreise
Otorhabdot	1	Otorhabdot
Otofett	3	
Pros (unreadable)	11	
Pros bromol	364	Monocelididae n.g. n.sp.
**Rhabdocoela: Dalytyphloplanidae (20 spp.)**		
Dalyblind	2	Dalytyph ng. nsp ("Orostylis ")
Dalylongphar	2	
Russelkopf rhabdit	12	
Russelkopf spirale	7	
Russelkopf kurzpenis	57	*Coronhelmis*
Russelkopf macro	260	Dumpy Typhloplanid
Promesostoma spirale	151	*Promesostoma* n.sp
Typhlotief	1	
**Rhabdocoela: Kalyptorhynchia (26 spp.)**		
Cinciaugi	3	Cinci augi
Cinciblind	13	Squarenose
Placo augi	6	*Placorhynchus doei*
Eukalypto spitz	23	*Placorhynchus doei* ?
Eukalyptozange	1	Eukalypto zange
Eukalyptoriese	1	EukalyptoRiese
Eukalyptospirale	125	
Eukalyptokompli	73	
Eukalyptoschrag	1	EukalyptoShrag
Gnathoschwanz	1	Gnathoschwanzi
Gnathospitz	1	*Drepanorhynchides hastatus*
Schizocarcharodo	135	
Schizoblindmacro	35	Schizochilus n.sp. "foxi"
Schizolongrussel	131	*Proschzorhynchus* n.sp. "shaunae"
Schizokurzrussel	13	
Schizofaroennis	4	*Carolinorhynchus follybeachensis*
Schizomesozoan	26	
Proschizospirale	1	Schizo4-belt
Schizoblindcompli	1	Schizoblind compli
Tylacoaugi	3	*Thylacorhynchus* "augi"
Tylacoschwanzi	72	*Thylacorhynchus* "schwanzi"
Carcharodo klein	45	Carcharodo klein
Carcharodo compli	6	Carcharodo compli
Karkinoaugi	84	*Lehardyia alleithoros*
Cheliplanilla schwanz	11	*Cheliplanilla* n.sp.

Cheliplana ohne schwanz	1	*Cheliplana* “Not BCOC"
Not matched to a Rieger species from EI		*Lehardyia* sp2
Not matched to a Rieger species from EI		*Karkinorhynchus* n.sp "ESTPG"
Not matched to a Rieger species from EI		Karkino2 small proboscis
Not matched to a Rieger species from EI		STL brown unknown (Proschizo)
Not matched to a Rieger species from EI		STL Schizo (Proschizo)
Not matched to a Rieger species from EI		Gracile Schizo
Not matched to a Rieger species from EI		Tiny Eukalypt
Not matched to a Rieger species from EI		Blind Cheli October
Not matched to a Rieger species from EI		*Coelogynopora* (Long Litho)
Not matched to a Rieger species from EI		*Diopisthoporus* "rubra"
Not matched to a Rieger species from EI		*Diopisthoporus gymnopharynx*
Not matched to a Rieger species from EI		*Microstomum* "gross"
Not matched to a Rieger species from EI		Pseudostomidae 4-eyes
Not matched to a Rieger species from EI		*Carcharodorhynchus* 99
Not matched to a Rieger species from EI		Schizo-2-belt
Not matched to a Rieger species from EI		Oto3
Not matched to a Rieger species from EI		*Diopisthoporus lofolitis*
Not matched to a Rieger species from EI		Schizo-2-belt
Not matched to a Rieger species from EI		ParotoGerade
Not matched to a Rieger species from EI		*Praeconvoluta* cf. *tigrina*
Not matched to a Rieger species from EI		*Hesiolicium* cf. *inops*
Not matched to a Rieger species from EI		*Cystiplana* cf. *rubra*
Not matched to a Rieger species from EI		Gliderband oto
Not matched to a Rieger species from EI		Proschizo tertius
Not matched to a Rieger species from EI		Proschizo knobby2
